# TonEBP recognizes R-loops and initiates m6A RNA methylation for R-loop resolution

**DOI:** 10.1093/nar/gkaa1162

**Published:** 2020-12-11

**Authors:** Hyun Je Kang, Na Young Cheon, Hyun Park, Gyu Won Jeong, Byeong Jin Ye, Eun Jin Yoo, Jun Ho Lee, Jin-Hoe Hur, Eun-A Lee, Hongtae Kim, Kyoo-young Lee, Soo Youn Choi, Whaseon Lee-Kwon, Kyungjae Myung, Ja Yil Lee, Hyug Moo Kwon

**Affiliations:** Department of Biological Sciences, Ulsan National Institute of Science and Technology, Ulsan 44919, Republic of Korea; Department of Biological Sciences, Ulsan National Institute of Science and Technology, Ulsan 44919, Republic of Korea; Department of Biological Sciences, Ulsan National Institute of Science and Technology, Ulsan 44919, Republic of Korea; Department of Biological Sciences, Ulsan National Institute of Science and Technology, Ulsan 44919, Republic of Korea; Department of Biological Sciences, Ulsan National Institute of Science and Technology, Ulsan 44919, Republic of Korea; Department of Biological Sciences, Ulsan National Institute of Science and Technology, Ulsan 44919, Republic of Korea; Department of Biological Sciences, Ulsan National Institute of Science and Technology, Ulsan 44919, Republic of Korea; UNIST-Optical Biomed Imaging Center (UOBC), Ulsan National Institute of Science and Technology, Ulsan 44919, Republic of Korea; Center for Genomic Integrity, Institute for Basic Science, Ulsan 44919, Republic of Korea; Department of Biological Sciences, Ulsan National Institute of Science and Technology, Ulsan 44919, Republic of Korea; Center for Genomic Integrity, Institute for Basic Science, Ulsan 44919, Republic of Korea; Center for Genomic Integrity, Institute for Basic Science, Ulsan 44919, Republic of Korea; Department of Biological Sciences, Ulsan National Institute of Science and Technology, Ulsan 44919, Republic of Korea; Center for Genomic Integrity, Institute for Basic Science, Ulsan 44919, Republic of Korea; Department of Biological Sciences, Ulsan National Institute of Science and Technology, Ulsan 44919, Republic of Korea; Center for Genomic Integrity, Institute for Basic Science, Ulsan 44919, Republic of Korea; Department of Biological Sciences, Ulsan National Institute of Science and Technology, Ulsan 44919, Republic of Korea

## Abstract

R-loops are three-stranded, RNA–DNA hybrid, nucleic acid structures produced due to inappropriate processing of newly transcribed RNA or transcription-replication collision (TRC). Although R-loops are important for many cellular processes, their accumulation causes genomic instability and malignant diseases, so these structures are tightly regulated. It was recently reported that R-loop accumulation is resolved by methyltransferase-like 3 (METTL3)-mediated m6A RNA methylation under physiological conditions. However, it remains unclear how R-loops in the genome are recognized and induce resolution signals. Here, we demonstrate that tonicity-responsive enhancer binding protein (TonEBP) recognizes R-loops generated by DNA damaging agents such as ultraviolet (UV) or camptothecin (CPT). Single-molecule imaging and biochemical assays reveal that TonEBP preferentially binds a R-loop via both 3D collision and 1D diffusion along DNA *in vitro*. In addition, we find that TonEBP recruits METTL3 to R-loops through the Rel homology domain (RHD) for m6A RNA methylation. We also show that TonEBP recruits RNaseH1 to R-loops through a METTL3 interaction. Consistent with this, TonEBP or METTL3 depletion increases R-loops and reduces cell survival in the presence of UV or CPT. Collectively, our results reveal an R-loop resolution pathway by TonEBP and m6A RNA methylation by METTL3 and provide new insights into R-loop resolution processes.

## INTRODUCTION

R-loops are three-stranded nucleic acid structures consisting of a DNA-RNA hybrid and a single-stranded (ss) DNA. R-loops have pleiotropic functions essential for eukaryotic physiology; they are important for chromosome segregation in mitosis, immunoglobulin class switching, DNA replication and repair, and transcription (1–3). Moreover, these structures have biological relevance in regulating gene expression and specialized rearrangement events (4,5). R-loops are enriched at promoters and terminator in poly A-positive genes, suggesting that they might have gene regulatory functions (3). However, R-loop accumulation can be a significant threat to genomic stability in several ways. Firstly, the fork structure at the extremities of R-loops can be cleaved by nucleotide excision repair proteins to generate double-stranded (ds) DNA breaks (DSBs). Secondly, R-loops cause transcription-replication collision (TRC), resulting in stalling and collapse of replication forks and the production of one-ended DSBs, both of which are substrates for chromosome translocations (2,5). R-loop accumulation is also associated with a variety of diseases involving genomic instability, including myelodysplastic syndromes, neurodegenerative diseases, and cancers such as Ewing's sarcoma (5–7). Given their potential to cause genomic instability, R-loops must be tightly regulated in cells. Although many factors have been identified for R-loop generation and resolution (8–11), it is still unknown how R-loops are intrinsically recognized and removed.

N6-methyladenosine (m6A) RNA methylation is a common and reversible RNA modification that post-transcriptionally directs many important processes of RNA. It is critical for cellular activities, such as T cell homeostasis and inflammatory, antitumor immune, and DNA damage responses (12). m6A RNA modification is co-transcriptionally catalyzed by the methyltransferase complex composed of methyltransferase-like 3 (METTL3), METTL14 and Wilms tumor 1-associated protein (WTAP) (13–15). Recently, m6A RNA methylation was identified as a novel factor for DNA polymerase kappa (Pol κ) recruitment to DNA damage sites (16). The modification occurs within 2 min at ultraviolet (UV) damage sites, raising the possibility of its role in the DNA damage response. UV-induced accumulation of m6A mRNA at damage sites is mediated by METTL3, which is also recruited to UV-damaged sites. Moreover, METTL3 deficiency is associated with reduced DNA repair capacity following UV exposure. Recent work showed that m6A RNA methylation on R-loops was required for R-loop removal under physiological conditions (17). ATM-dependent phosphorylation of METTL3 promotes to catalyze m6A modification in R-loops to stimulate homologous recombination (HR) repair in YTHDC1 dependent manner at DNA DSB (18). However, how R-loops are recognized and METTL3 is recruited to DNA damage sites and how m6A RNA methylation by METTL3 contributes to R-loop resolution at damaged DNA are still unknown.

Tonicity-responsive enhancer binding protein (TonEBP) is a pleiotropic transcriptional regulator, either an activator or a suppressor depending on individual genes, that regulates genes in a variety of physiological and pathological conditions (19). It binds a specific DNA sequence called TonE with affinity in the range of 50 nM, which is much lower than typical DNA binding proteins with affinities <1 nM (20). Interestingly, the crystal structure of the DNA binding domain of TonEBP reveals a protein ring encircling dsDNA, raising the possibility that TonEBP has role in DNA surveillance. It was recently reported that TonEBP senses bulky DNA adducts and modifies ubiquitination of proliferating cell nuclear antigen (PCNA), leading to DNA damage repair (21,22). The change in PCNA ubiquitination is achieved by dynamic interactions of TonEBP with the E3 ubiquitin-protein ligase SHPRH and ubiquitin specific peptidase 1 (21). Thus, TonEBP is capable of sensing DNA damage and orchestrating signaling events via interactions with multiple enzymes.

In the present study, we analyzed proteins that interact with TonEBP by combining immunoprecipitation and mass spectrometry and identified METTL3 among over 450 binding candidates. We then investigated the role of the TonEBP-METTL3 interaction in the setting of UV-induced R-loop resolution. We reveal that TonEBP senses damage-induced R-loops and recruits METTL3 for m6A RNA methylation to promote R-loop resolution by RNaseH1. Furthermore, using *in vitro* biochemical and single-molecule assays, we demonstrated that TonEBP identifies R-loops via both 3D collision and 1D diffusion along DNA.

## MATERIALS AND METHODS

### Cells and reagents

HEK293T and U2OS cells were cultured in Dulbecco's modified Eagle's medium (DMEM) supplemented with 10% fetal bovine serum (FBS; Thermo Fisher Scientific Inc., Waltham, MA, USA), 100 U/ml penicillin, and 100 μg/ml streptomycin (GE Healthcare Life Sciences, Logan, UT, USA). Cells were maintained at 37°C in an incubator with 5% of CO_2_. Antibodies used for immunoblotting or immunoprecipitation were obtained from various companies. Cells were transfected with Lipofectamine 2000 or Lipofectamine RNAimax (Invitrogen, Carlsbad, CA, USA). siRNA duplexes were purchased from Integrated DNA Technologies (Coralville, IA, USA).

### Tandem affinity purification and mass spectrometry analysis

Detailed method is provided in supplementary information.

### Immunofluorescence, microscopy and image analysis

Cells were plated on LabTek chamber slides (Thermo Fisher Scientific) and incubated for 1 day before fixation with 100% methanol at 20°C for 30 min. For chromatin-bound proteins, cells were pretreated with 0.5% Triton X-100 for 2 min before fixation. For laser microirradiation, UVA laser (55 mW) irradiation was performed by means of a Palm MicroBeam laser microdissection workstation. The fixed cells were stained with the appropriate primary antibodies overnight at 4°C. After washes with 0.05% Triton X-100, Alexa Fluor–conjugated secondary antibodies were added and incubated for 1 h. The stained cells were mounted. Alexa Fluor 488-, 568- and 633-conjugated secondary antibodies were purchased from Invitrogen. To exclude the unexpected effects of nucleolar structures on R-loop, the spread method of R-loop immunostaining was performed as previously described (23,24). The detailed protocol was described in Supplementary Information.

### Immunoprecipitation

For preparation of lysates for immunoprecipitation, cells were washed three times with ice-cold PBS and lysed in RIPA buffer as described previously (25). An appropriate antibody was added to lysates and incubated overnight at 4°C, followed by incubation with Protein A/G Sepharose beads (GE Healthcare Sciences). After extensive washing with RIPA lysis buffer, complexes were eluted and analyzed by immunoblotting.

### S9.6 IP

Cells were trypsinized, washed with 1X PBS, and resuspended in 25 ml of 1× PBS. Cells were crosslinked in 1% formaldehyde (Pierce), quenched with 0.125 M glycine, and washed twice in 1× PBS containing protease inhibitor (PI; Roche). The cells were lysed with the lysis buffer (50 mM HEPES pH 7.9, 140 mM NaCl, 1 mM EDTA, 10% glycerol, 0.5% NP-40, 0.25% Triton X-100) with protease inhibitor cocktail and chromatin was sonicated in the shearing buffer (0.1% SDS, 1 mM EDTA, 10 mM Tris–HCl pH 8.1) on a ultrasonicator (Covaris) to an average size of 1 kb. Washed Protein A/G sepharose beads (Pierce) were used to pre-cleared chromatin for 2 h. 10 μg of chromatin fraction was mixed with either 20 μg of S9.6 antibody or 20 μg mouse IgG and incubated overnight at 4°C. Pre-washed protein A/G sepharose beads were then added to the chromatin/antibody mixture for 2 h. After washing three times with the binding buffer (10 mM NaPO4 pH 7.0, 140 mM NaCl, 0.05% Triton X-100), bound beads were boiled in 30 μl 5× sample buffer (10% SDS, 500 mM DTT, 50% Glycerol, 250 mM Tris–HCl pH 6.8 and 0.5% bromophenol blue dye) and loaded on a 4–20% gradient gel (Bio-Rad).

### Immunoblotting

Cell lysis for protein extraction was performed as described elsewhere (26). Protein concentration was measured with the BCA Protein Assay System (Pierce, Rockford, IL, USA). Equal amounts of protein from each sample were separated by SDS PAGE and immunoblotted using specific primary antibodies (Supplementary Table S1). Horseradish peroxidase (HRP)-conjugated mouse, rabbit and goat secondary antibodies were used for detection. The antigen–antibody binding was detected by means of enhanced chemiluminescence western blotting detection reagents (GE Healthcare Life Sciences).

### PLA (proximity ligation assay)

For the proximity ligation assay (PLA), cells were pre-extracted with cold 0.5% NP-40 for 3 min on ice. Cells were then fixed with 4% PFA/PBS for 15 min, washed 3 times with 1× PBS and blocked for 1 h at room temperature (RT, 25°C) with 2% BSA/PBS. Cells were then incubated with a primary antibody overnight at 4°C. Cells were washed 3 times in 1× PBS and incubated in a pre-mixed solution of PLA probe anti-mouse minus and PLA probe anti-rabbit plus (Sigma) for 1 h at 37°C. The Duolink In Situ Detection Reagents (Green) were then used to perform the PLA reaction according to the manufacturer's instructions. Slides were mounted in Duolink In Situ Mounting Medium with DAPI and imaged on a Zeiss Axioscope at 40×. The number of PLA foci inside a nucleus was quantified using the Image J software (NIH). Since the PLA is a sensitive assay and might generate artifacts if not properly controlled, PLA assays with single antibody were carried out as a negative control under the same condition as above. We did not observe any PLA signals with single antibody (Supplementary Figure S16).

### Cell survival analysis

HEK293T or U2OS cells were plated in triplicate in 96-well plates, and MTT assays were performed according to the manufacturer's protocol (Bio-Rad). The absorbance at 490 nm was measured using a multi-well plate reader (Tecan M200).

### The molecular combing assay

HEK293T cells were labeled for 30 min with 50 μM CldU (C6891, Sigma-Aldrich) followed by 30 min of labeling with 250 μM IdU (I7125, Sigma-Aldrich). To measure DNA replication rates, the cells were embedded in low-melting agarose (161-3112, Bio-Rad) followed by DNA extraction. To stretch the DNA fibers, 22 × 22 mm silanized coverslips (Genomic Vision) were dipped into the DNA solution for 13 min and pulled out at a constant speed (300 μm/s) using Molecular Combing System (Genomic Vision MCS-001). The coverslips were baked at 60°C for 4 h and incubated with 4 N HCl for denaturation. CldU- and IdU-labeled tracts were detected by 2-h incubation at RT with a rat anti-BrdU antibody (dilution 1:100 detects BrdU and CldU; Abcam 6326) and a mouse anti-BrdU antibody (1:10, detects BrdU and IdU; Becton Dickinson 347580). The slides were fixed in 4% paraformaldehyde in 1× PBS and incubated for 1 h at RT with an Alexa Fluor 488–conjugated goat anti-rat IgG antibody (dilution 1:100, A21208; Molecular Probes/Thermo Fisher) or an Alexa Fluor 568–conjugated goat anti-mouse IgG antibody (dilution 1:100, A21124; Molecular Probes/Thermo Fisher). Finally, the coverslips were mounted with the ProLong Gold Antifade Reagent (Molecular Probes) and stored at −20°C. DNA fibers were imaged under a Carl Zeiss microscope with Axio Observer 7 & ApoTome 2 (Motorized Fluorescence Microscope with Grid Projection) and 63× objective. For each experiment, a total of 200 DNA fibers were analyzed, and the length of DNA fibers was measured in Adobe Photoshop.

### Separation of nuclear and chromatin-bound fractions

The nuclear fraction was extracted using the Nuclear Extraction Kit (Pierce). The chromatin-bound fraction was extracted as described previously (27).

### Buffer for *in vitro* assays

Unless otherwise mentioned, TonEBP buffer for *in vitro* assays is 10 mM HEPES pH 7.5, 1 mM DTT and 5% glycerol supplemented with different concentrations of NaCl.

### Purification of TonEBP

Yc1 domain was subcloned between NdeI and XhoI sites of pET19b derivative. The protein construct had triplex FLAGs (3xFLAG) at N-terminus and ten histidines (His_10_) at C-terminus. The plasmid containing Yc1 was transformed into Rosetta (DE3) or BL21 (DE3). Cells were grown in 1 L LB media supplemented with 50 μg/ml carbenicillin at 37°C and 235 rpm. When OD_600_ reached about 0.6, proteins were expressed by adding 1 mM IPTG (isopropyl β-d-1-thiogalactopyranoside), and cells were further grown at 37°C for 3 h. After harvested, cells were resuspended in 15 ml of resuspension buffer (50 mM HEPES pH7.5, 200 mM NaCl, 5 mM Imidazole, 1 mM DTT and 1× protease inhibitor (Halt, Thermo Fisher Scientific) and then snap-frozen in liquid N_2_ for –80°C storage until use. After cells were thawed, cells were lysed by sonication and then clarified by ultracentrifugation at 27 000 × g for 20 min. The clarified lysates were loaded onto 15 ml bed volume of Talon gravity column. After washing the Talon gravity column with five column volumes of washing buffer (50 mM HEPES pH 7.5, 200 mM NaCl, 1 mM DTT and 5 mM Imidazole), the proteins were eluted with elution buffer (50 mM HEPES pH 7.5, 200 mM NaCl, 1 mM DTT and 300 mM Imidazole). Pooled fractions were dialyzed against storage buffer (10 mM HEPES pH 7.5, 200 mM NaCl, 1 mM DTT and 5% glycerol) and then stored at –80°C.

### Electrophoretic mobility shift assay for R-loop binding of Yc1

R-loop substrates for electrophoretic mobility shift (EMSA) were prepared by following the previous protocol (23). R-loop oligo1 labeled with Cy3, R-loop oligo2, and R-loop RNA were mixed at equi-molar ratio in 10 mM Tris–HCl pH 7.5 and 100 mM NaCl and then were heated at 95°C, followed by slow cooling to 23°C (Supplementary Table S2). Duplex DNA was formed by annealing Homoduplex and R-loop oligo2 (Supplementary Table S2). To test the R-loop formation, 30 nM of R-loop construct or duplex DNA was incubated with 230 nM S9.6 (ENH001, Kerafast) in 25 mM Tris–HCl pH 8.0 and 150 mM NaCl at 23°C for 30 min. The supershift by S9.6 was measured by 5% non-denaturing PAGE in 0.5× TBE (Supplementary Figure S1A). For the binding affinity of Yc1 to different types of DNA substrates, we made bubble, RNA–DNA hybrid, fork, and ssDNA in addition to R-loop and homoduplex. R-loop oligo1 was used as ssDNA, and bubble structure was made by hybridization between R-loop oligo1 and R-loop oligo2 without RNA. Fork structure was prepared by annealing Fork1 and Fork2. RNA–DNA hybrid was formed by annealing hybrid DNA and hybrid RNA (Supplementary Table S2). 30 nM of DNA substrate was incubated with Yc1 at different concentrations in TonEBP buffer (10 mM HEPES pH 7.5, 1 mM DTT and 5% glycerol) with 50 mM NaCl at 23°C for 2 h. All the reactants were run on 5% non-denaturing PAGE in 0.5× TBE and then Cy3 or Cy5 fluorescence signal was imaged by Typhoon 2000 (GE Healthcare).

### Single-molecule DNA curtain assay

Detailed method is provided in supplementary information.

### Quantification and statistical analysis

All statistical analyses were performed by Student's *t* test. In figures, n means the number of biological replicates.

## RESULTS

### TonEBP interacts with METTL3

**Figure 1.**
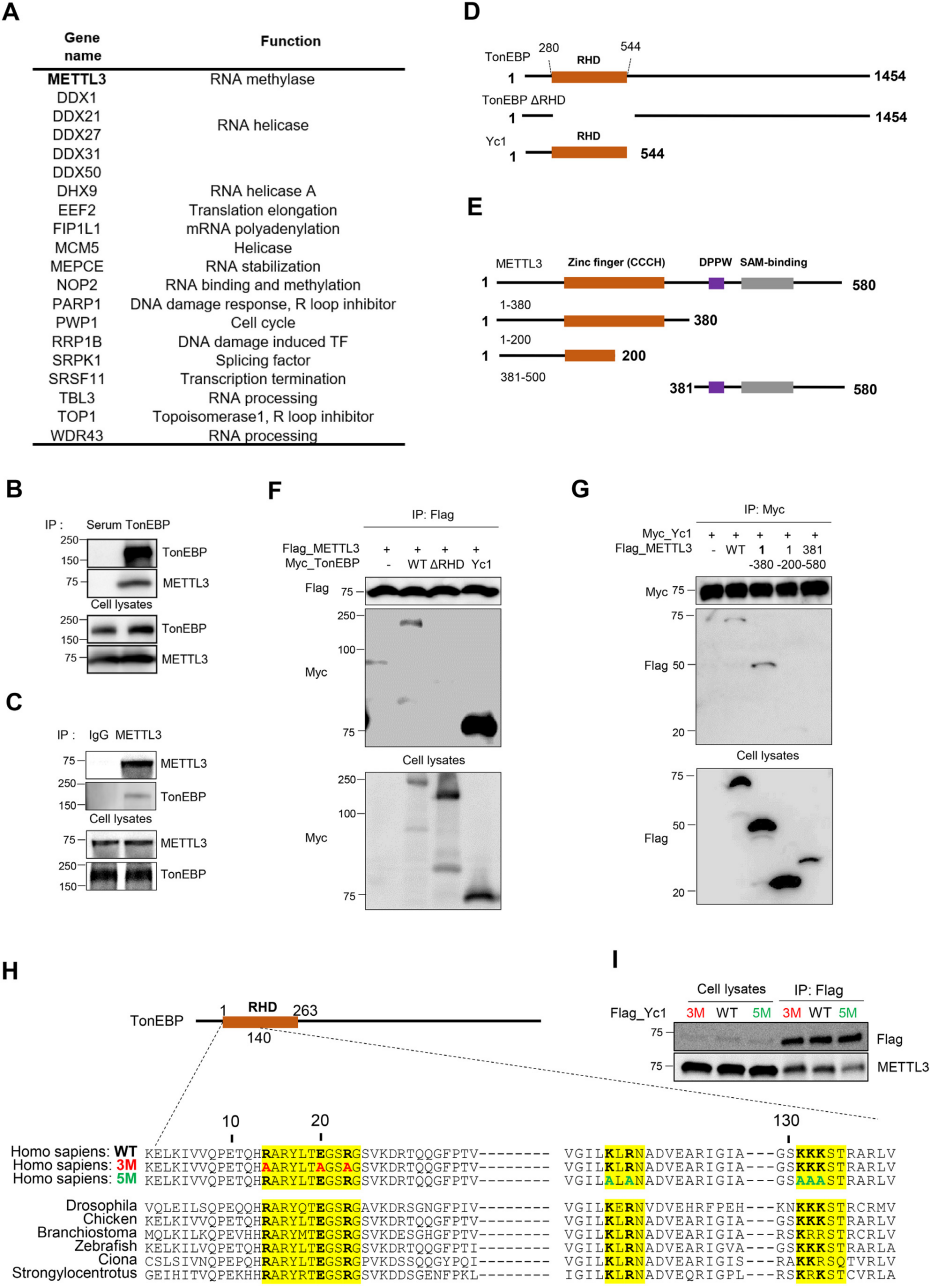
TonEBP interacts with METTL3 and m6A methylase. (**A**) The TonEBP interactome includes METTL3 and R-loop-related proteins. (**B**) HEK293T cell lysates were immunoprecipitated with normal serum (Serum) or anti-TonEBP antibody (TonEBP). Precipitates and cell lysates were blotted for TonEBP and METTL3. (**C**) Cell lysates were immunoprecipitated with normal rabbit IgG (IgG) or anti-METTL3 IgG (METTL3). (**D**) Domain structures of human TonEBP (WT), and deletion proteins ΔRHD and Yc1. (**E**) Domain structures of human METTL3 (WT) and deletion proteins 1–380, 1–200 and 381–580. (**F**) Cells were transfected with plasmids expressing Flag-METTL3 together with Myc-tagged TonEBP (WT), ΔRHD, or Yc1. After 24 h, cell lysates were prepared and immunoprecipitated using anti-FLAG antibody. (**G**) Cells were transfected with plasmids expressing Myc-Yc1 together with Flag-tagged METTL3 (WT), 1–380, 1–200 or 381–500 and immunoprecipitation was performed with Myc antibody 24 h later. (**H**) Amino-acid sequence alignment of the highly conserved and charged regions of TonEBP from seven species. 3M is a mutant Yc1 in which R, E and R were all exchanged to A shown in red and 5M is another mutant where K, R, and the three Ks were all replaced by A shown in green. (**I**) Cells were transfected with a plasmid expressing Flag-tagged Yc1, 3M or 5M. Cell lysates were prepared and immunoprecipitated with anti-FLAG antibody after 24 h incubation.

Combining tandem affinity purification and mass spectrometry, we identified proteins that interact with the N terminus of TonEBP (TonEBP Yc1 domain: Yc1), which encompasses the entire Rel homology domain (RHD) of TonEBP. The list contains 20 proteins involved in R-loop resolution, including METTL3 and RNA helicases (Figure 1A). METTL3 catalyzes the post-transcriptional methylation of internal adenosine residues in eukaryotic mRNAs, forming m6A, which is critical for R-loop resolution (14,17). Therefore, we investigated the interaction between TonEBP and METTL3 and their roles in R-loop resolution. We first confirmed that TonEBP physically binds METTL3 using mutual co-immunoprecipitation assays (Figure 1B and C). To determine the domains involved in the TonEBP-METTL3 interaction, serial deletion mutants of TonEBP and METTL3 were generated (Figure 1D and E). Co-immunoprecipitation with the deletion mutants of TonEBP revealed that the RHD of TonEBP interacts with the zinc finger domain (201-380 a.a.) of METTL3 (Figure 1F and G). Because the RHD of TonEBP has conserved amino acids sequences, we produced two TonEBP mutant proteins carrying point mutations (3M and 5M) at the highly conserved and charged amino acids (Figure 1H). 3M is a mutant of Yc1 in which R, E and R are all replaced by A shown in red and 5M is another mutant where K, R and the three Ks are all exchanged with A shown in green. The 5M mutant of Yc1 reduced interaction with METTL3 compared to wild-type and the 3M mutant of Yc1 (Figure 1I). Collectively, these results demonstrate that the TonEBP RHD interacts with METTL3.

### TonEBP is recruited to damaged DNA where it induces m6A RNA methylation

**Figure 2.**
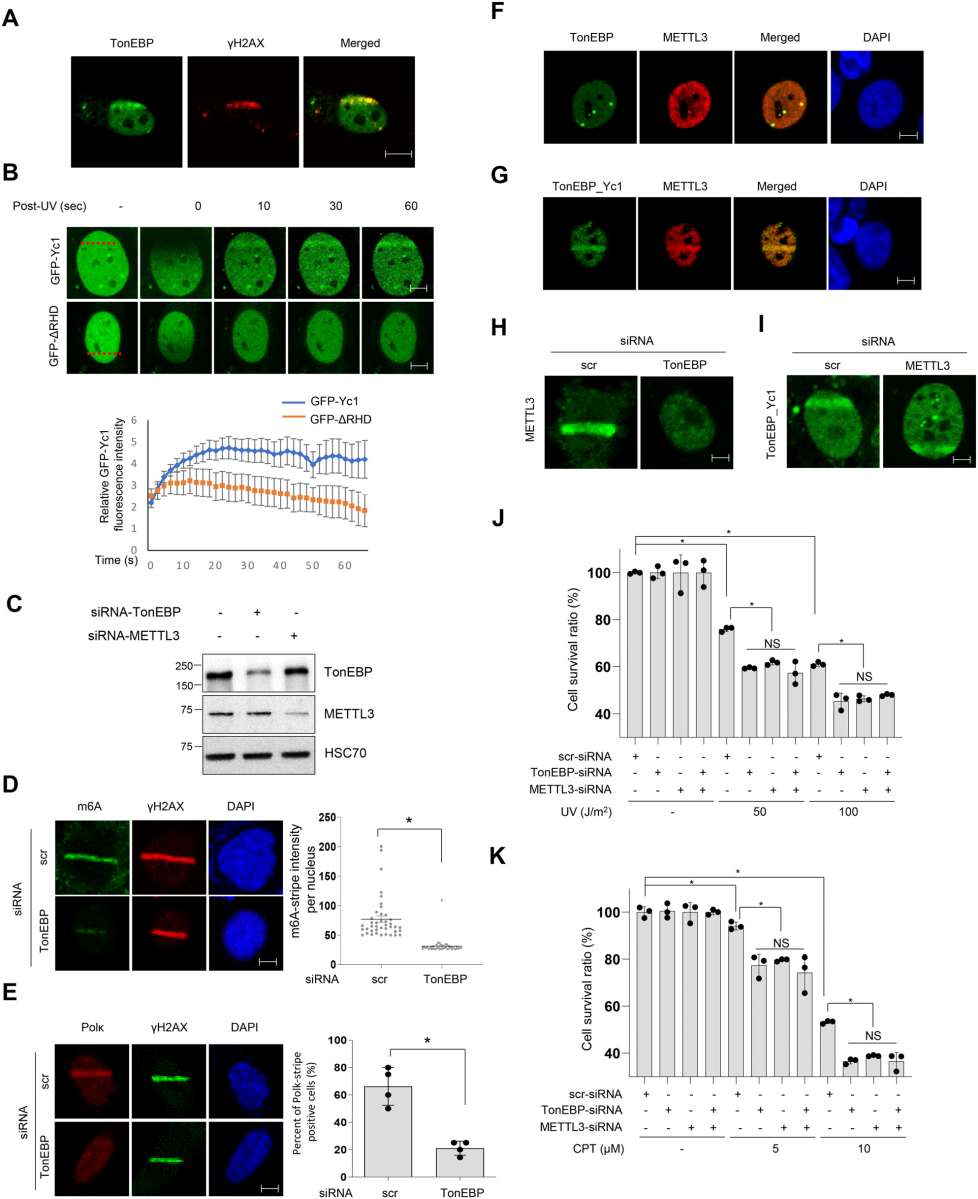
TonEBP is recruited to DNA damage sites and induces m6A RNA methylation. (**A**) U2OS cells were subjected to laser microirradiation followed by double staining for TonEBP and γH2AX. Representative images are shown (scale bar: 2 μm). (**B**) Top: cells were transfected with plasmids expressing GFP-tagged Yc1 (upper panel) or ΔRHD (lower panel). After 24 h, cells were laser microirradiated at the positions marked by a red dashed line (left). Green fluorescence images were taken 0–60 s after irradiation. Bottom: the fluorescence intensity in the microirradiated area at each time point was determined from 20 cells. Mean ± SD, *P* < 0.01 from 10 to 60 s. (**C**) Immunoblot analysis of the knockdown efficiency of siRNA in U2OS cells. Heat shock cognate 71 kDa protein (HSC70) was used for loading control. (**D**) Cells were transfected with scrambled siRNA (scr) or TonEBP-targeting siRNA (TonEBP) for 24 h and then subjected to laser microirradiation. After 2 min, immunostaining was performed for m6A and γH2AX. Left: representative images of a nucleus in each condition. Right: the laser stripe intensity was determined for 40 cells in each condition. Mean ± SD, *P < 0.01. (**E**) siRNA-transfected cells were subjected to laser microirradiation followed by incubation for 2 min and immunostained for Pol κ and γH2AX. Left: representative images. Right: percentage of Pol κ laser stripe-positive cells from 10 cells. Mean ± SD, *n* = 4. *P < 0.01. (**F**) Cells were immunostained for full-length TonEBP and METTL3; a representative set of images are shown. (**G**) Cells were transfected with two plasmids expressing GFP-TonEBP_Yc1 and mCherry-METTL3 for 24 h. Green and red fluorescence images were obtained 2 min after laser microirradiation. (**H**) siRNA-transfected cells were transfected a second time with a plasmid expressing GFP-METTL3, and green fluorescence images were taken 2 min after laser microirradiation. (**I**) siRNA-transfected cells were transfected a second time with a plasmid expressing GFP-Yc1, and green fluorescence images were taken after laser microirradiation. (**J**) Cells were transfected with various combinations of scrambled-siRNA (−), TonEBP-targeting siRNA, and METTL3-targeting siRNA for 24 h as indicated. Cells were subjected to 0–100 J/m^2^ of UV irradiation, and the cell survival percentage after 24 h was measured. Mean ± SD, *n* = 3; *P < 0.01; NS, *P* > 0.05. (**K**) Cells were transfected as (**J**) and treated with 0–10 μM CPT for 24 h, and then the cell survival percentage after 24 h was measured.

The interaction of TonEBP with METTL3 and other R-loop related proteins raises the possibility that TonEBP plays a role in DNA repair by regulating R-loops. Indeed, we previously reported that TonEBP is recruited to DNA damage sites generated by methyl methanesulfonate where TonEBP facilitates DNA repair by fork remodeling (21). METTL3-mediated m6A RNA methylation is required for the UV-induced DNA damage response, and co-transcriptional R-loops are resolved by METTL3-mediated m6A RNA methylation (16,17). Because TonEBP directly binds METTL3, we investigated TonEBP’s activities at UV-induced DNA damage sites including R-loops and its role in m6A RNA methylation. We observed localization of TonEBP to damaged DNA when cells were treated with laser microirradiation, which causes multiple types of DNA damage including R-loops (28,29). Endogenous TonEBP was colocalized with PCNA and γH2AX, the markers for DNA damage in response to laser microirradiation (Supplementary Figure S1 and Figure 2A). Yc1 including the RHD of TonEBP was translocated to the microirradiated region of the nucleus within 10 s, while ΔRHD of TonEBP was not (Figure 2B). Our results suggest that TonEBP is capable of locating UV-irradiated sites in the early stage in an RHD-dependent manner. We next assessed whether TonEBP played a role in the UV-induced m6A RNA methylation. In both immunocytochemistry and dot blot analyses, TonEBP knockdown significantly reduced UV-induced m6A RNA methylation without changes in γH2AX, indicating that TonEBP is required for the m6A methylation (Figure 2C and D, and Supplementary Figure S2). To confirm the decrease in m6A RNA methylation, we examined Pol κ, which is a direct downstream target of m6A RNA (16). TonEBP knockdown reduced the UV-induced recruitment of Pol κ, consistent with the role of TonEBP in m6A RNA methylation. When TonEBP expression was suppressed, Pol κ recruitment to DNA damage sites was significantly reduced (Figure 2E).

Given the finding that TonEBP binds METTL3, we examined their colocalization on DNA damage sites. In U2OS cells not treated with DNA damaging agents, TonEBP spontaneously formed foci that overlapped with METTL3 foci (Figure 2F). TonEBP and METTL3 colocalization was pronounced at microirradiated DNA damage sites (Figure 2G). Interestingly, METTL3 recruitment was TonEBP-dependent (Figure 2H) but Yc1 (TonEBP) recruitment was not dependent on METTL3 (Figure 2I). This suggests that DNA damage is recognized by TonEBP, which then recruits METTL3. We also monitored the cell survival response to UV or CPT after individual or combined deletion of TonEBP and METTL3. Lack of TonEBP and/or METTL3 did not cause a significant difference in cell survival upon UV irradiation or CPT treatment (Figure 2J and K), suggesting that TonEBP and METTL3 are epistatic for m6A RNA methylation at DNA damage sites. Taken together, our results indicate that TonEBP promotes DNA damage-induced m6A RNA methylation as an upstream factor for METTL3, which results in Pol κ recruitment to DNA damage sites.

### TonEBP preferentially binds R-loops

**Figure 3.**
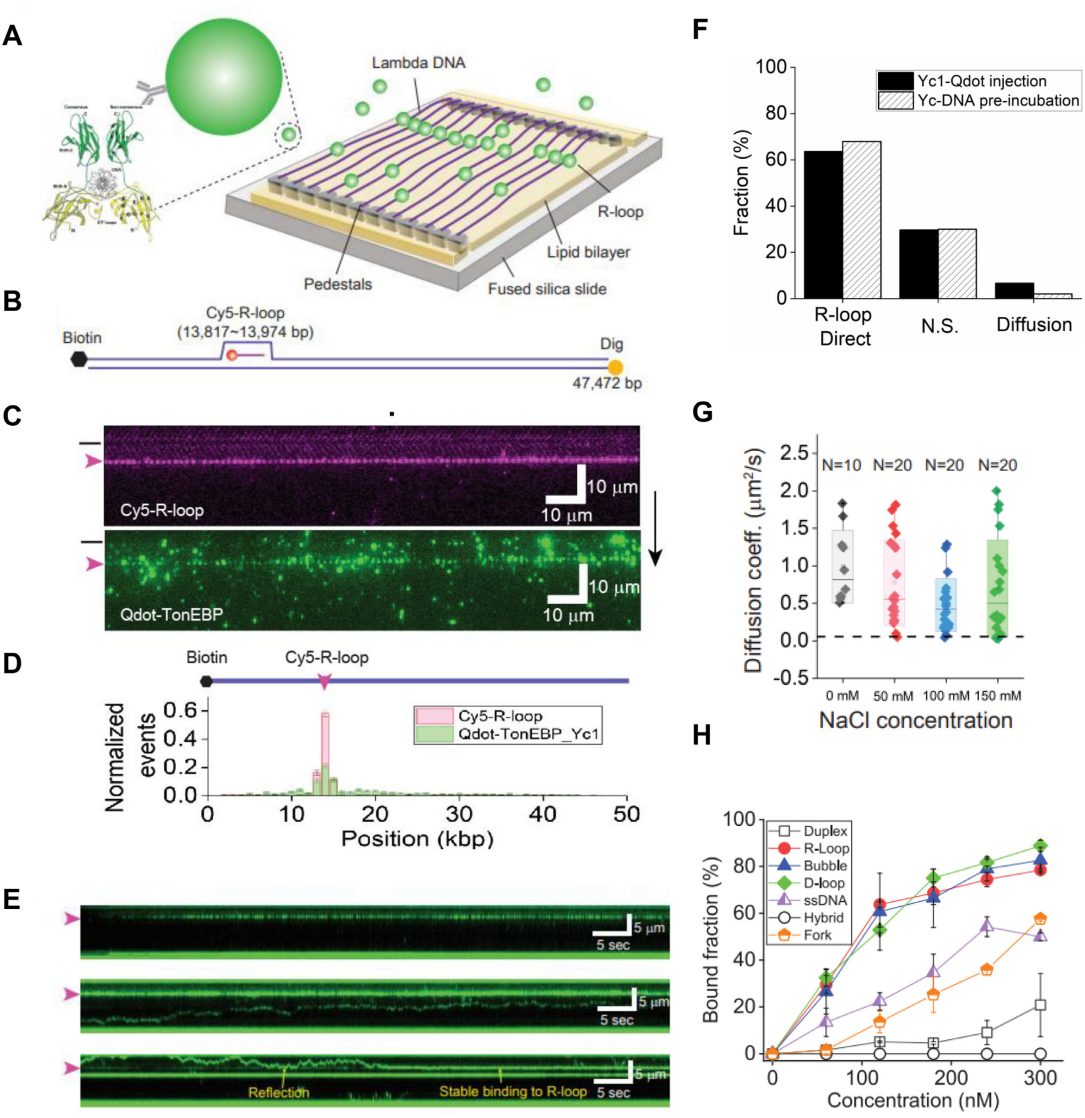
TonEBP preferentially binds R-loops *in vitro*. (**A**) Schematic of the double-tether DNA curtain, in which one end of λ-DNA was anchored on the lipid bilayer via biotin-streptavidin linkage and the other end is attached to chromium pedestals via digoxigenin and its antibody binding. TonEBP structure is adopted from Protein Data Bank (PDB: 1IMH). (**B**) Schematic of λ-DNA (λ-I3) containing a Cy5-labeled R-loop, which is the same as Supplementary Figure S4B except that the other end is labeled with digoxigenin (yellow circle). (**C**) Two-color images of a single-tether DNA curtain. (Top) Fluorescence image of Cy5-R-loop (magenta) in the DNA curtain (Supplementary Figure S4) and (bottom) fluorescence image of Qdot-labeled Yc1 (green). The black bar and magenta arrowhead left to the images indicate the barrier position and R-loop location, respectively. The black arrow right of the figure denotes the flow direction. (**D**) Binding distribution of Cy5-R-loop (magenta) and Qdot-Yc1 (green). Both peaks overlapped at the R-loop position. (**E**) Top: kymograph displaying Yc1 binding to R-loop after flow was off when a maximum amount of Yc1 arrived at a DNA curtain. Yc1 directly bound to R-loops without diffusion. Middle: kymograph showing diffusion of Yc1 on λ-DNA without an R-loop. Yc1 was pre-incubated with the λ-DNA, and then the DNA curtain was formed with the λ-DNA. Bottom: kymograph showing R-loop binding of Yc1 via 1D diffusion. Yc1 was pre-incubated with the λ-DNA, and then the DNA curtain was formed with the λ-DNA. Two Yc1 molecules were associated with a single R-loop. (**F**) Quantification of Yc1 behavior in a double-tether DNA curtain. Filled black: Yc1 and Qdot were pre-incubated and then injected into DNA curtain (total number of molecules: 160). Dashed line: Yc1 and λ-DNA were pre-incubated and then a double-tether DNA curtain was formed. Yc1 was labeled by Qdot inside the flowcell (total number of molecules: 165). (**G**) Diffusion coefficients according to salt concentrations. The black dashed line represents the theoretical limit (0.057 μm^2^/s, Supplementary Information) of rotational motion along a DNA helix. *N* represents the number of molecules. (**H**) Quantified data for EMSA of Yc1 with different types substrates (Supplementary Figure S3B) showing the binding affinity of Yc1 to different types of DNA substrates. Yc1 preferentially binds ssDNA region of R-loop, D-loop and bubble.

Since TonEBP recruits METTL3 at DNA damage sites and promotes m6A RNA modification, we hypothesized that TonEBP would recognize R-loops. To test this, we investigated the interaction between TonEBP and R-loops *in vitro* using purified Yc1. A Cy3-labeled R-loop construct was formed using synthesized oligonucleotides and confirmed by gel shift with S9.6 antibody (Supplementary Information, Supplementary Figure S3A, and Table S2). The R-loop binding of Yc1 was tested by electrophoretic mobility shift assay (EMSA), which showed that Yc1 has higher binding affinity to R-loops than to homoduplex DNA (Supplementary Figure S3B). We also performed single-molecule DNA curtain assay to elucidate the Yc1 and R-loop interaction. Yc1 was pre-incubated with lambda DNA (λ-DNA) containing Cy5-labeled R-loops at specific positions to form single-tether DNA curtains (Supplementary Figure S4A and B) (30). Yc1 tagged with 3xFLAG was labeled with anti-FLAG-conjugated quantum dots (Qdots) inside the flowcell. Cy5-labeled R-loop and the Qdot-labeled Yc1 were fluorescently visualized, both of which overlapped (Figure 3C and D). When we reversed the λ-DNA, the fluorescence signals of Cy5 and Qdot were also reversed and still overlapped (Supplementary Figure S4C and D). We also tested λ-DNA containing a different R-loop construct placed at different position (Supplementary Figure S4E). Yc1 also colocalized with the R-loop, indicating that the preferential binding of Yc1 to R-loop in DNA curtain is sequence-independent. Taken together, our results demonstrate that Yc1 preferentially binds R-loops.

We next investigated how Yc1 identified R-loops using double-tether DNA curtain assays (Figure 3A and B). We injected 5 nM Yc1 labeled with Qdot into the double-tether DNA curtains, and the engagement of Qdot-Yc1 with DNA was observed (Figure 3E, top). Quantitatively, most Yc1 (68%) directly and stably bound to R-loops, while few Yc1 (2%) diffused along DNA besides nonspecific bindings (30%) (Figure 3F). These results strongly suggest that the Yc1 most likely recognized R-loop through 3D-collision. Based on EMSA and DNA curtain data above, the binding affinity of TonEBP to the duplex is very low. To scrutinize the diffusion of Yc1, we pre-incubated Yc1 with the λ-DNA for 2 h. In the double-tether DNA curtain, diffusive population of Yc1 increased (6.6%), while most Yc1 already occupied R-loop (63%) (Figure 3E middle and bottom and F). We observed that diffusive Yc1 was settled at the R-loop after it reflected several times from the R-loop, at which another Yc1 was already bound (Figure 3E bottom). Our results demonstrated that Yc1 senses R-loops through 1D diffusion in addition to 3D collision, and two Yc1 molecules can bind a single R-loop. We also calculated diffusion coefficients of Yc1 according to salt concentration (Figure 3G and Supplementary Figure S5). The diffusion coefficient did not vary with increased ionic strength, suggesting that Yc1 diffuses along DNA *via* sliding not hopping (31). In the sliding mode, the protein might rotate along the DNA helices. We calculated the theoretical limit of the diffusion coefficient for rotational motion (black dashed line in Figure 3G and Supplementary Information), which is markedly smaller than the diffusion coefficients without rotation, suggesting that TonEBP slides without rotation along the DNA helix (32). It was also reported that TonEBP specifically interacts with the consensus sequence (5′-TGGAAANNYNY-3′) of the nuclear factor of activated T-cells (NFAT) family. Although R-loops containing λ-DNA have eight NFAT consensus sites, we observed no pauses or stalls at these sequences (Supplementary Figure S5D).

We further examined how TonEBP interacts with R-loops using different types of DNA substrates (Figure 3H and Supplementary Figure S3). Interestingly, TonEBP bound R-loop, bubble, and D-loop structures with the same affinity, whereas it did not bind RNA–DNA hybrid. TonEBP also bound ssDNA with lower affinity than R-loop. In addition, the binding affinity of Yc1 to a fork structure of Y-shape is lower than that of R-loop. Collectively, these results demonstrated that TonEBP recognizes the displaced ssDNA of R-loop. The difference of binding affinity between displaced ssDNA of R-loop and ssDNA might result from the diffusion-away of TonEBP through open end of ssDNA.

### TonEBP depletion causes R-loop accumulation and transcription-replication conflicts

**Figure 4.**
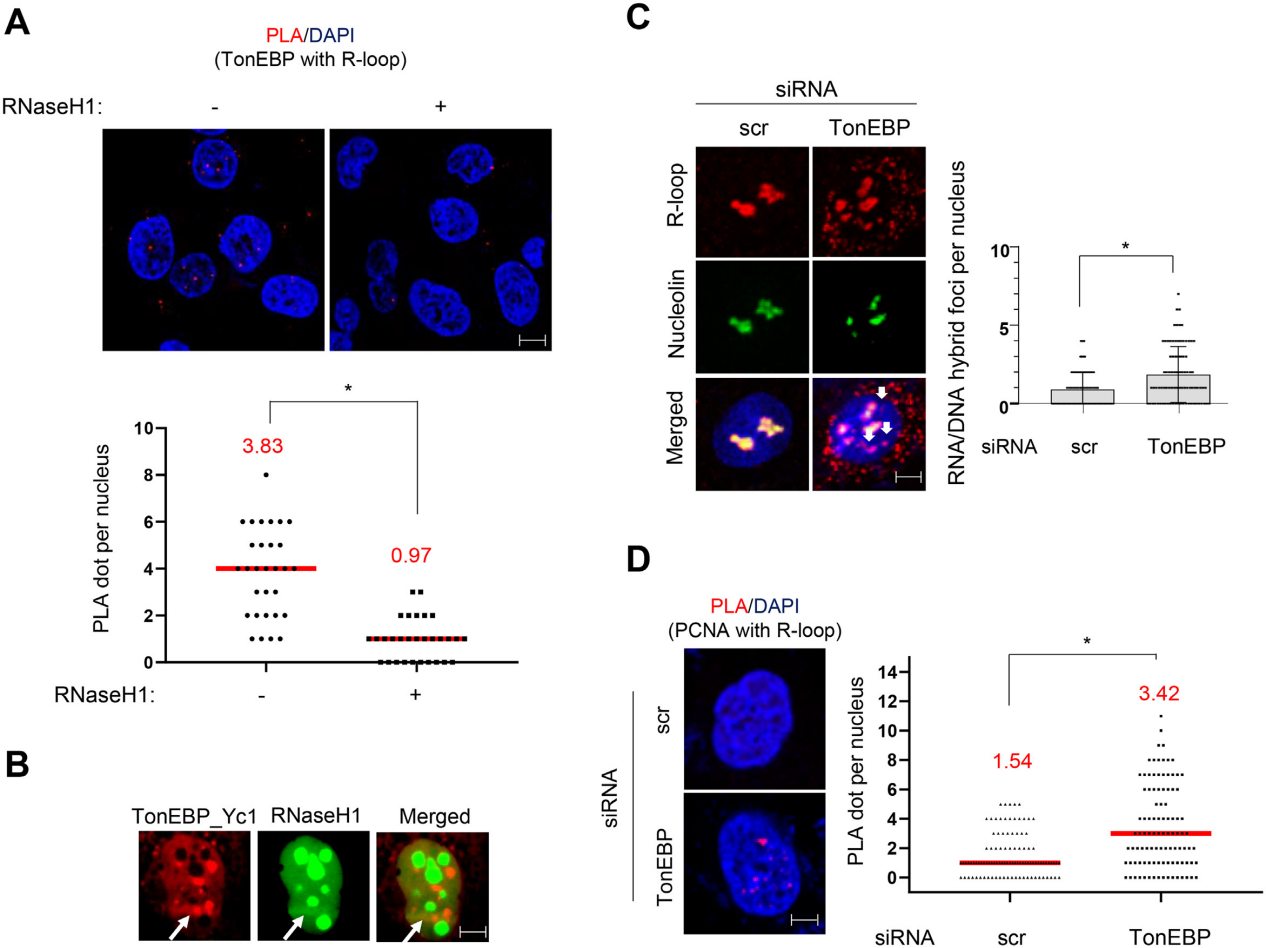
TonEBP colocalizes with R-loops and TonEBP depletion induces R-loop accumulation *in vivo*. (**A**) PLA between TonEBP and R-loops in U2OS cells. Top: representative images in the absence (left) and presence (right) of overexpressed catalytically-active RNaseH1. Bottom: number of PLA foci per nucleus calculated from 30 cells. Red line represents mean value, which is also denoted above in red. (**B**) Cells were transfected with plasmids expressing mCherry-TonEBP_Yc1 and catalytically-active GFP-RNaseH1 for 24 h. Cells were then subjected to laser microirradiation (arrow), and fluorescence images were taken 2 min later. Representative images of single nucleus are presented. (**C**) U2OS cells were transfected with scrambled siRNA (scr) or TonEBP-targeting siRNA (TonEBP) for 48 h. Cells were fixed and immunostained for S9.6 and nucleolin. (Left) Representative images. (Right) The S9.6 foci per nucleus after subtracting nucleolar signals (white arrows) were counted from 100 nuclei. Mean ± SD, *P < 0.01. (**D**) PLA between R-loops (S9.6) and PCNA in siRNA-transfected U2OS cells. Left: representative images for each condition. Right: number of PLA foci per nucleus calculated from 100 cells. Red line represents mean value, which is also denoted above in red.

We next asked whether TonEBP also binds R-loops *in vivo*. We performed proximity ligation assays (PLA) with TonEBP antibody and S9.6 antibody that can detect R-loops. PLA signals were observed in the nucleus but there was no PLA signal when catalytically-active RNaseH1 was overexpressed, indicating that PLA signals generated between TonEBP and S9.6 were produced at R-loops under physiological conditions (Figure 4A). We also examined TonEBP binding to R-loops formed at DNA damage sites using laser microirradiation. TonEBP overlapped with catalytically-active RNaseH1 at the irradiated sites (Figure 4B).

Given that TonEBP interacts with METTL3 and binds R-loops, we hypothesized that TonEBP would influence R-loop levels *in vivo*. Immunocytochemistry analysis with S9.6 antibody showed enhanced S9.6 intensity when TonEBP was depleted (Figure 4C and Supplementary Figure S15A). R-loop accumulation was previously shown to lead to TRC (33). In the PLA between PCNA and S9.6 or between PCNA and RNA polymerase II (RNAP2), TonEBP knockdown increased the signals, suggesting that TonEBP depletion increases TRCs due to R-loop accumulation (Figure 4D and Supplementary Figure S3C). The elevated TRCs in the TonEBP-depleted cells were associated with reduced fork velocity and lower levels of active, chromatin-bound PCNA throughout S-phase (Supplementary Figure S6A and B). In addition, EdU incorporation measured by a non-antibody azide/alkyne reaction between EdU and a fluorescent probe was reduced by TonEBP knockdown (Supplementary Figure S6C). Accordingly, TonEBP knockdown reduced cell proliferation (Supplementary Figure S6D). To confirm that slow proliferation and replication stress following TonEBP depletion were due to R-loop accumulation, catalytically-active RNaseH1 was overexpressed while TonEBP was depleted. Catalytically-active RNaseH1 overexpression recovered DNA synthesis and cell proliferation induced by TonEBP depletion (Supplementary Figure S6E and F). Collectively, the results demonstrate that TonEBP deficiency increases R-loop accumulation, followed by TRC-induced replication stress and defective cell proliferation.

### m6A RNA methylation is specific to R-loops at DNA damage sites

**Figure 5.**
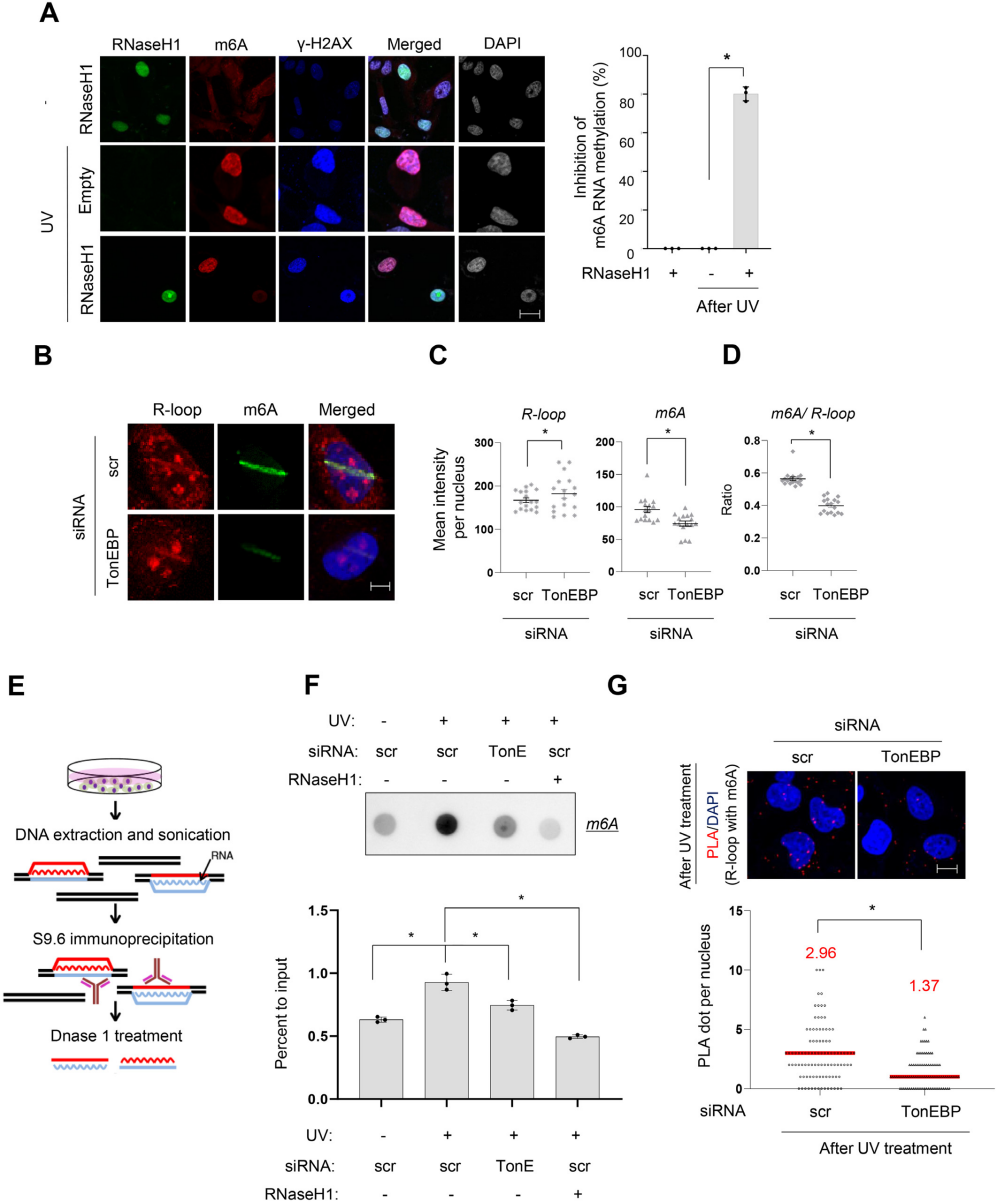
TonEBP is required for m6A RNA methylation on R-loops. (**A**) U2OS cells transfected with a plasmid containing catalytically-active GFP-RNaseH1 or no RNaseH1 (empty) were subjected to UV irradiation (0 or 60 J/m^2^) as indicated and fixed and double stained for m6A and γH2AX 2 min later. Left: representative fluorescence images of GFP, m6A, and γH2AX from the same slide treated without or with UV. Right: when the experiment was performed without GFP-RNaseH1 transfection, 100% of cells showed m6A RNA methylation after UV treatment (i.e. 0% inhibition of m6A RNA methylation). When 10 RNaseH1-positive cells were examined for m6A methylation from the experiments shown at left, only ∼20% cells showed m6A RNA methylation (i.e. ∼80% inhibition of m6A RNA methylation). Mean ± SD, *n* = 3, *P < 0.01. (**B**) siRNA-transfected cells were subjected to laser microirradiation and then immunostained for R-loop (S9.6) and m6A RNA (m6A). Representative images of nuclei for each siRNA are presented. (**C**) Mean fluorescence intensities per microirradiation stripe for S9.6 and m6A were measured from at least 15 nuclei from three independent experiments shown in (B). Mean ± SD. *P < 0.01. (**D**) The ratio of m6A to S9.6 was calculated from the experiments in panel (B). Mean ± SD. *P < 0.01. (**E**) Schematic of DNA:RNA hybrid immunoprecipitation (DRIP). (**F**) Cells transfected with scrambled siRNA (scr) or TonEBP-targeting siRNA (TonE) were treated without or with UV as indicated. DRIP was performed and analyzed by m6A RNA dot blotting. Top: representative dot blot. Bottom: the signal in each dot was corrected by RNA content to obtain percent to input. Mean ± SD, *n* = 3, *P < 0.01. (**G**) siRNA-transfected cells were treated with UV followed by PLA for m6A and S9.6. Top: representative images. Bottom: the numbers of PLA dots per nucleus were counted in at least 250 nuclei from three independent experiments. Mean ± SD, *P < 0.01.

m6A RNA methylation occurs at laser microirradiated sites where R-loops are also formed (Figures 2D and 4B). Thus, we investigated whether RNA methylation is specific to R-loops at damage sites. We measured DNA damage-induced m6A RNA methylation of poly A (+) mRNA in chromatin-bound mRNA and in the nucleoplasm and cytoplasm. m6A RNA methylation was detected on chromatin-bound mRNA (Supplementary Figure S7A and B), suggesting that m6A RNA methylation due to DNA damage mainly occurs in the chromatin. We observed that R-loop colocalized with m6A methylation in the nucleus (Supplementary Figure S7C). In addition, when catalytically-active RNaseH1 was overexpressed, DNA damage-induced m6A RNA methylation was reduced by up to 80% based on m6A immunocytochemistry (Figure 5A).

We then examined if TonEBP is involved in m6A RNA methylation of R-loops at damage sites. Immunocytochemistry analysis of R-loops after TonEBP knockdown increased the level of R-loops generated by laser microirradiation, whereas the level of m6A RNA methylation was substantially decreased (Figure 5B–D). To confirm the immunocytochemistry data, we performed dot blot analyses of m6A in R-loops immunoprecipitated with S9.6 antibody. For S9.6 immunoprecipitation, cellular extracts underwent DNA extraction followed by fragmentation, and R-loops were immunoprecipitated with anti-S9.6 antibody. RNA strands from the R-loops were released by DNase1 treatment, and only poly A (+) mRNAs were used for m6A dot blots (Figure 5E). TonEBP depletion reduced DNA damage-induced m6A RNA methylation at R-loops (Figure 5F). Consistently, DNA damage-induced PLA signals between S9.6 and m6A were decreased by TonEBP knockdown (Figure 5G).

**Figure 6.**
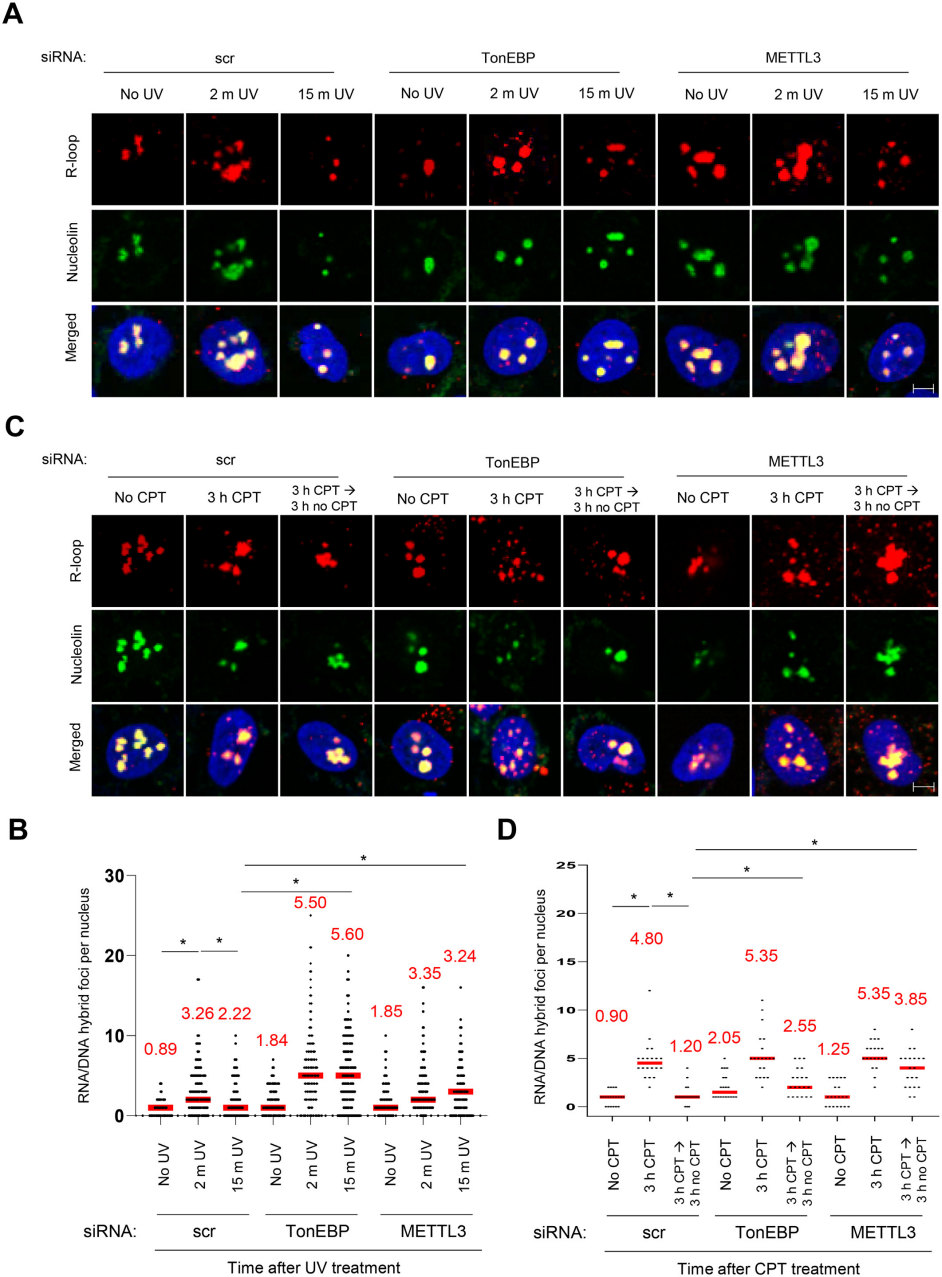
TonEBP resolves R-loops. (A and B) Cells transfected with siRNA as indicated were irradiated with UV (60 J/m^2^) and analyzed at 2 or 15 min after UV-irradiation. R loop and nucleoli were visualized by immunostaining (**A**). R-loop foci per nucleus were counted from >80 nuclei in three independent experiments (**B**). Mean ± SD, *P < 0.01. Mean values are shown as numbers. (**C** and **D**) siRNA-transfected cells were either untreated (no CPT) or treated with 10 μM CPT for 3 h and analyzed immediately or after 3 h further incubation in fresh media without CPT.

Next, we asked whether TonEBP and METTL3 would promote R-loop resolution at damaged sites. We measured time-course R-loop levels after UV- or CPT-induced DNA damage. Knockdown of TonEBP or METTL3 curtailed the efficiency of R-loop resolution, suggesting that TonEBP and METTL3 are required for promoting R-loop resolution at damaged sites via m6A RNA methylation (Figure 6 and Supplementary Figure S15B). Furthermore, we investigated the role of m6A RNA methylation in R-loop resolution. Since m6A RNA methylation is induced by METTL3, we tested catalytically-inactive METTL3, in which D395 and W398 were replaced by A (Supplementary Figure S8A) (34). RNaseH1 was not recruited to laser-microirradiated sites when the mutant METTL3 was expressed, showing that m6A RNA methylation by METTL3 is necessary for R-loop resolution (Supplementary Figure S8B and C).

### The RHD of TonEBP is important for R-loop resolution

**Figure 7.**
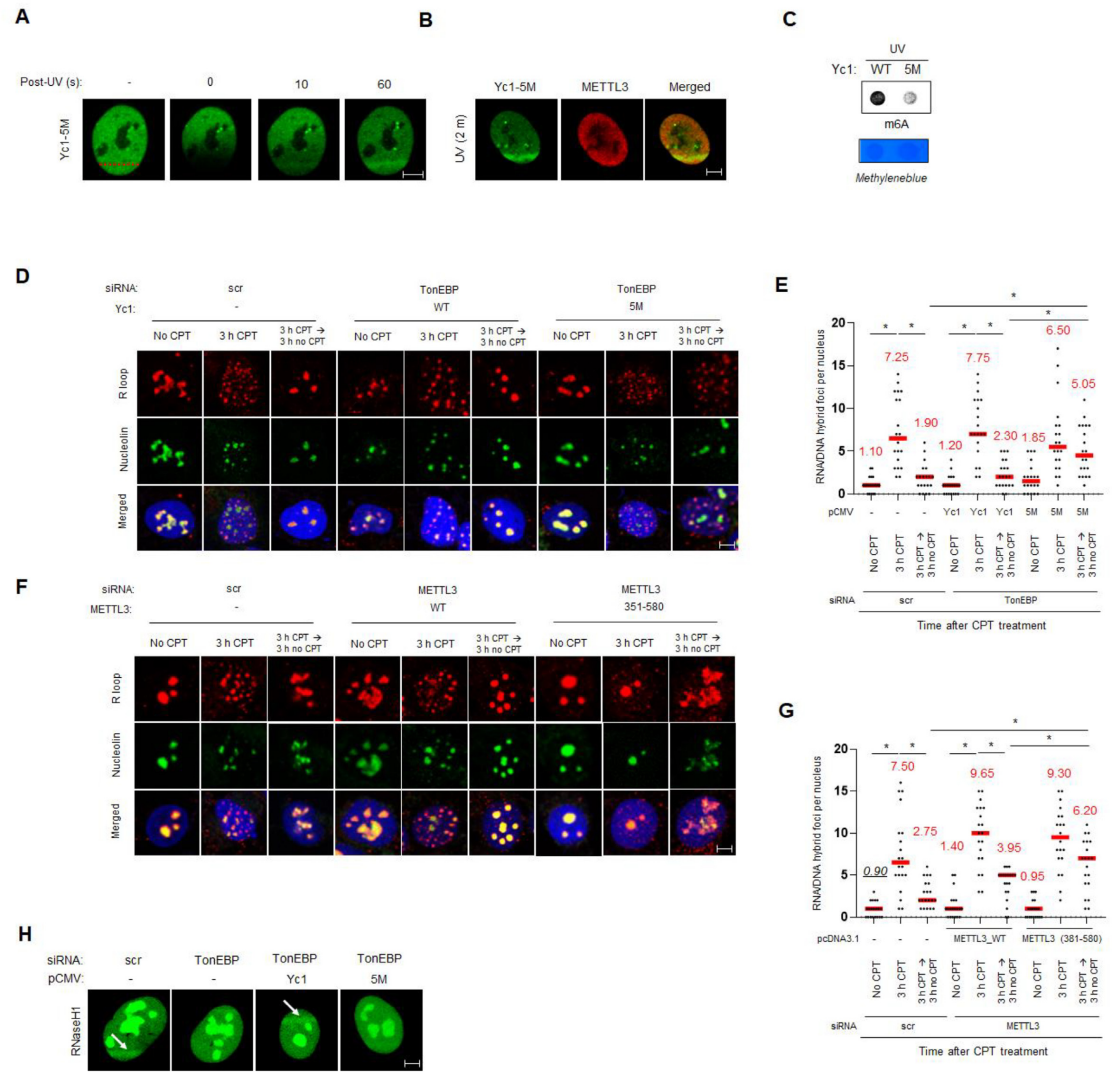
Interaction between TonEBP and METTL3 is required for R-loop resolution. (**A**) U2OS cells were transfected with plasmids expressing GFP-5M Yc1 (Yc1-5M) for 24 h. Cells were then laser microirradiated, and representative images were obtained 0, 10 and 60 s later. (**B**) U2OS cells were transfected with two plasmids expressing GFP-Yc1-5M and mCherry-METTL3. Cells were subjected to laser microirradiation, and representative fluorescence images were obtained 2 min later. (**C**) U2OS cells were transfected with plasmids expressing Yc1 (WT) or 5M mutant Yc1 (5M) for 24 h. m6A dot blotting was performed after UV treatment. Cell lysates were subjected to dot blot assays with anti-m6A antibody. (**D**, **E**) siRNA-transfected U2OS cells were transfected a second time with a plasmid expressing Yc1 (WT) or 5M. S9.6 and nucleoli were immunostained. (**D**) Representative images. (**E**) The numbers of S9.6 foci per nucleus were counted from >20 nuclei from three independent experiments. Mean ± SD, *P < 0.01. (F, G) siRNA-transfected cells were transfected a second time with a plasmid expressing METTL3 (WT) or 351–580. (**F**) Representative images. (**G**) The numbers of S9.6 foci per nucleus were counted from >20 nuclei from three independent experiments. (**H**) U2OS cells were double transfected with siRNA and pCMV expressing Yc1 and 5M in the indicated combinations. The cells were transfected a second time with a plasmid expressing catalytically-active GFP-RNaseH1. Representative GFP fluorescence images were taken 2 min after laser microirradiation (arrows).

The RHD of TonEBP is involved in both R-loop binding and the METTL3 interaction. We inspected the role of RHD of TonEBP in R-loop resolution (Figures 1 and 3). First, we checked whether the 5M mutant that weakly binds METTL3 localized to DNA damage sites. The 5M mutant of Yc1 recruited to laser microirradiated sites (Figure 7A). Then we investigated the effect of RHD on R-loop resolution. The 5M mutant could not rescue METTL3 recruitment to DNA damage sites (Figure 7B), m6A RNA methylation (Figure 7C), and R-loop resolution, (Figure 7D and E) upon UV irradiation or CPT treatment. Pol κ was not recruited to DNA damage sites when 5M mutant was expressed, suggesting that TonEBP-mediated pol κ recruitment depends on METTL3 (Supplementary Figure S9). In addition, the RHD deletion mutant and 5M mutant of Yc1 could not rescue cell survival defects (Supplementary Figure S10) following UV irradiation or CPT treatment. Similarly, (381-580) mutant METTL3, which cannot interact with TonEBP, could not rescue R-loop resolution (Figure 7F and G) or cell survival defects (Supplementary Figures S11) upon UV irradiation or CPT treatment.

Under physiological conditions, m6A RNA methylation in R-loops leads to their resolution due to recruitment of an m6A binding protein named YTHDF2 (17). Catalytically-active RNaseH1 is recruited to R-loop in response to UV irradiation (Figure 4), so we examined whether m6A RNA methylation played a role in this event. RNaseH1 was co-immunoprecipitated with TonEBP and colocalized with TonEBP at irradiated sites (Supplementary Figure S12 and Figure 4B). However, when TonEBP expression was suppressed, RNaseH1 recruitment to the microirradiated sites disappeared (Figure 7H). Interestingly, while the complementation of Yc1 increased RNaseH1 recruitment, the 5M mutant that is incapable of interaction with METTL3 did not (Figure 7H). These results indicate that the interaction between TonEBP and METTL3 is important for R-loop resolution *via* RNaseH1 recruitment.

### TonEBP-mediated m6A methylation occurs at damage-induced R-loops

**Figure 8.**
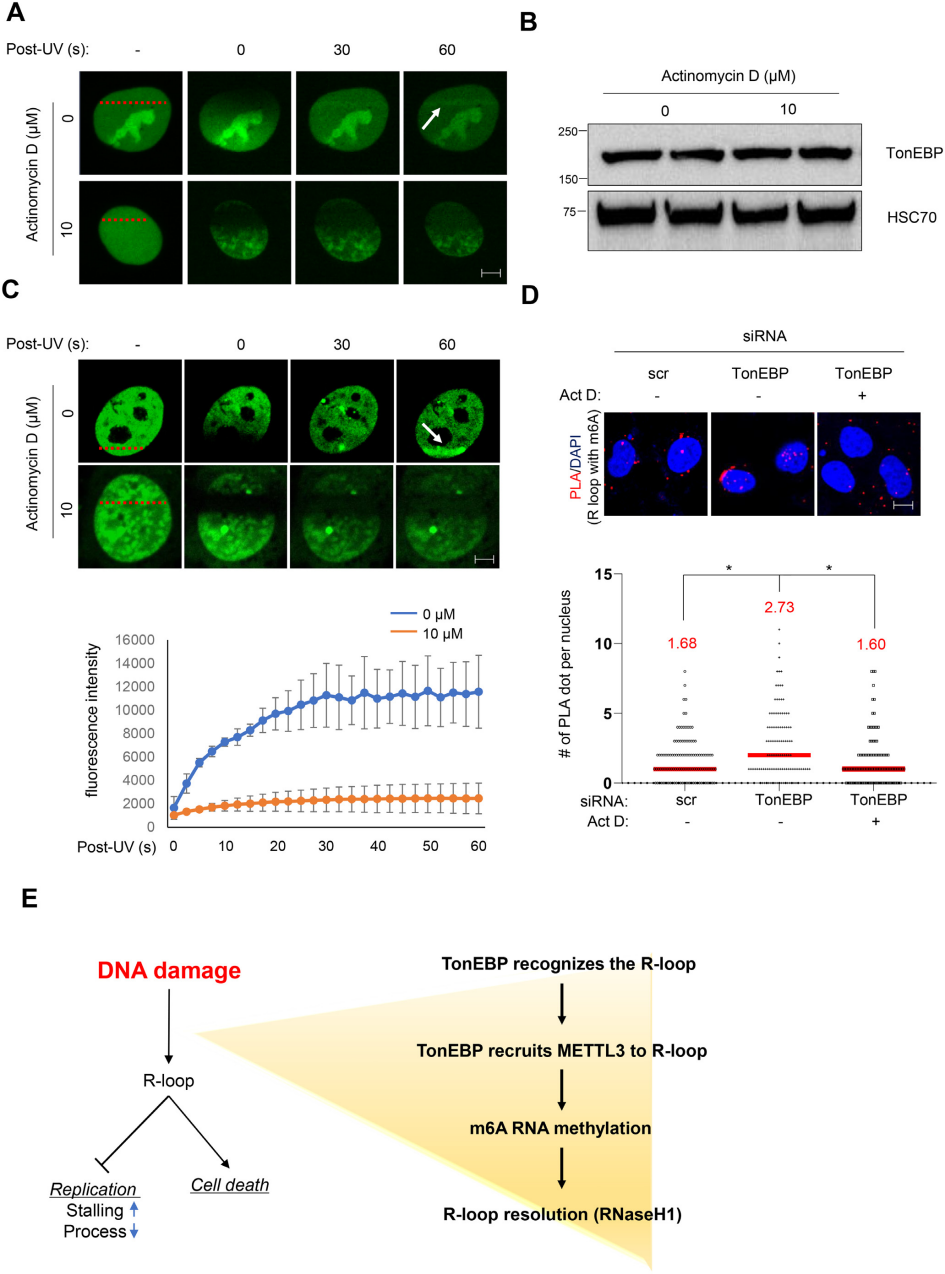
Blockade of R-loop formation reduces TonEBP recruitment and m6A RNA methylation at DNA damage sites. (**A**) U2OS cells transfected with a plasmid expressing catalytically-inactive GFP-RNaseH1 were pretreated for 10 min with 0 or 10 μM actinomycin D. Green fluorescence images were taken before (−) and 0 to 60 s after microirradiation at the positions indicated by red bars. Representative images are shown for each condition. White arrow indicates the microirradiation stripe. (**B**) Cells were pretreated with 0 or 10 μM actinomycin D for 10 min and immunoblotted for TonEBP and HSC70. (**C**) Cells were transfected with a plasmid expressing GFP-Yc1 and treated as in (A). Top: representative images. White arrow indicates the microirradiation stripe. Bottom: the fluorescence signal from each laser stripe was measured from 0 to 60 s as indicated. Mean ± SD, *n* = 10. *P* < 0.01 for all time points except 0 s. (**D**) siRNA-transfected cells were treated without (−) or with actinomycin D (Act D) (+) without any damaging agents. PLA was performed for S9.6 and m6A RNA. Representative images (top) and the numbers of PLA dots per nucleus (bottom) were counted from >50 nuclei in three independent experiments. Mean ± SD, *P < 0.01. (**E**) Model of TonEBP function in DNA damage-induced RNA methylation at R-loops.

Lastly, we examined whether TonEBP recruitment and m6A methylation would be reduced when R-loop formation was blocked using actinomycin D (28,35,36). To avoid unexpected change of protein expression level, cells were treated with high concentration of actinomycin D (10 μM) for a short time (10 min). R-loop formation (based on catalytically-inactive RNaseH1 recruitment) following laser microirradiation was completely blocked when cells were pretreated with actinomycin D for 10 min without changes in protein levels (Figure 8A and B).

GFP-Yc1 recruitment was also completely blocked by actinomycin D under the same conditions, consistent with the lack of TonEBP recruitment to R-loop (Figure 8C). The lack of TonEBP recruitment was associated with a marked reduction of m6A RNA methylation in response to UV irradiation (Supplementary Figure S13). Reduced R-loop formation and m6A RNA methylation were also observed in response to actinomycin D treatment under basal conditions (i.e. without UV) (Figure 8D). These findings demonstrate that TonEBP-METTL3-mediated m6A methylation occurs in R-loops.

Since YTHDF2 serves as a reader of m6A RNA methylation for R-loop resolution under the physiological condition, we asked if YTHDF2 plays the same role when DNA is damaged (17). We tested whether or not the recruitment of YTHDF2 to R-loop due to DNA damage is dependent on TonEBP by performing PLA experiments between R-loop and YTHDF2 when TonEBP was suppressed. Under the physiological condition, TonEBP knockdown slightly increased PLA signals. As shown in Figure 4C and Supplementary Figure S2, TonEBP depletion increased spontaneous R-loops and m6A methylation in R-loops, which was read by YTHDF2. In spite of TonEBP depletion, the m6A methylation in spontaneous R-loops seems to be caused by m6A methylation pathways other than TonEBP-METTL3. On the other hand, when cells were treated with CPT, the PLA signal significantly increased, whereas TonEBP depletion reduced PLA signals to the TonEBP-knockdown level under the physiological condition (Supplementary Figure S14). These results showed that the interaction between YTHDF2 and R-loop is dependent on TonEBP and YTHDF2 is on the TonEBP pathway. Conclusively, A6 in R-loop can be methylated by either TonEBP-dependent or independent pathway. But in the presence of DNA damage, m6A methylation via TonEBP-dependent pathway becomes be dominant.

## DISCUSSION

R-loops play important roles in cellular processes, but non-physiological R-loops are threats to genomic stability and must be removed (2,5,6,33). m6A RNA methylation is the most prevalent post-transcriptional modification and is involved in diverse RNA metabolic processes, but its role in R-loop resolution is poorly understood (37–39). A recent study reported that the m6A RNA methylation within R-loops is required for their resolution under basal conditions (17). Nevertheless, how R-loops generated by DNA damaging agents are resolved remains unclear. Here, we demonstrated that m6A methylation of RNA happened to R-loops in response to UV and CPT, implying that m6A RNA methylation is a general feature of R-loops. Surprisingly, we found that TonEBP, a transcriptional factor that regulates cellular osmotic pressure, serves as an upstream sensor to rapidly identify R-loops in response to UV within 10 s (Figure 8E). *In vitro* studies showed that TonEBP is extremely efficient in recognizing and binding R-loops, consistent with its ability to detect and bind R-loops *in vivo*. In addition, TonEBP directly binds METTL3 through the RHD domain, which is also important for R-loop recognition, and recruits METTL3 to the R-loop for m6A RNA methylation. TonEBP depletion not only results in decrease of m6A RNA methylation but also diminishes the interaction between R-loop and YTHDF2, which is a reader of m6A RNA methylation to eliminate R-loops. In addition, RNaseH1 is not recruited to damaged DNA when METTL3 is catalytically-inactive. The R-loop accumulation due to depletion of TonEBP causes replication stress and slow cell proliferation. Our results strongly suggest that the TonEBP-METTL3-m6A RNA methylation pathway is responsible for RNaseH1 recruitment and R-loop resolution. Thus, m6A RNA methylation in R-loops contributes to their resolution by removing RNA both under basal conditions and after UV exposure.

The crystal structure of the TonEBP RHD dimer bound to dsDNA is quite unusual for a transcriptional regulator in that the dimer completely encircles dsDNA (20). In addition, there is substantial space between the DNA surfaces and the protein ring. It has been suggested that TonEBP functions in DNA surveillance (22). We demonstrated that TonEBP (i.e. Yc1) binds R-loops with very high efficiency via 3D collision. This binding is far more efficient than its binding to dsDNA. Based on the crystal structure, inefficient TonEBP loading onto dsDNA is presumably due to the fact that it encircles DNA; however, once loaded, TonEBP diffuses along dsDNA until it binds to and stays in R-loops. In other words, TonEBP binding to R-loops occurs through dual search mechanisms: 3D collision and 1D diffusion. To our knowledge, it is extremely rare for a protein to employ a two-pronged mechanism like this. Nuclear TonEBP has a homogeneous signal as a GFP-fusion protein or in immunohistochemistry, suggesting that most nuclear TonEBP is not bound to DNA. When R-loops are formed, it is likely that TonEBP rapidly and efficiently loads onto R-loops using the dual search mechanism, leading to their m6A RNA methylation and resolution. Furthermore, our EMSA results with diverse types of DNA substrates demonstrated that TonEBP recognizes displaced ssDNA of R-loop. Likely, TonEBP also preferentially binds D-loop and bubble structures. Such preferential binding of TonEBP to the displaced ssDNA accounts for the dual search mechanism because the displaced ssDNA is accessible via either 3D collision or 1D diffusion along DNA. Moreover, our results imply that TonEBP serves as a general sensor to detect DNA damage such as R-loop, D-loop, or bubble.

In summary, our results demonstrate that TonEBP is an early sensor of R-loops. It loads and stays on R-loops, where it initiates a cellular pathway leading to m6A RNA methylation and R-loop resolution. The upstream function of TonEBP is likely to have widespread consequences since R-loop homeostasis is important in cell physiology and a variety of diseases.

## Supplementary Material

gkaa1162_Supplemental_File
