# Supplementary material for: TonEBP recognizes R-loops and initiates m6A RNA methylation for R-loop resolution
Source: Nucleic Acids Res. 2020 Dec 11;49(1):269–84. doi: 10.1093/nar/gkaa1162 (PMC7797050; doi:10.1093/nar/gkaa1162)
Supplement: gkaa1162_Supplemental_File [file gkaa1162_supplemental_file.docx]

**Supplementary Information for**

**TonEBP recognizes R-loop and initiates m6A RNA methylation for R-loop resolution**

Hyun Je Kang^1*^, Na Young Cheon^1*^, Hyun Park^1^, Gyu Won Jeong^1^, Byung Jin Ye^1^, Eun Jin Yoo^1^, Jun Ho Lee^1^, Jin-Hoe Hur^3^, Eun-A Lee^2^, Hongtae Kim^1,2^, Kyoo-young Lee^2^, Soo Youn Choi, Whaseon Lee-Kwon, Kyungjae Myung^1,2^, Ja Yil Lee^1,2,^, Hyug Moo Kwon^1^

1. School of Life Sciences, Ulsan National Institute of Science and Technology, Ulsan 44919, Republic of Korea

2. Center for Genomic Integrity, Institute for Basic Science, Ulsan 44919, Republic of Korea

3. UNIST-Optical Biomed Imaging Center (UOBC), Ulsan National Institute of Science and Technology, Ulsan 44919, Republic of Korea

**This file includes:**

**Ⅰ. Supplementary methods**

**Ⅰ.1.** Tandem affinity purification (TAP) and mass spectrometry analysis

**Ⅰ.2.** Single-molecule DNA curtain assay

**Ⅰ.3.** Immunofluorescence assay for R-loop without nucleolus

**Ⅰ.4.** References

**Ⅱ. Supplementary Figures**

**Ⅱ. 1.** Figure. S1. TonEBP co-localizes with PCNA in DNA damage sites.

**Ⅱ. 2.** Figure. S2. TonEBP induces UV-induced m6A RNA methylation.

**Ⅱ. 3.** Figure S3. TonEBP preferentially binds R-loop, especially the displaced ssDNA *in vitro*

**Ⅱ. 4.** Figure. S4. Single-molecule visualization of TonEBP and R-loop interaction *in vitro*

**Ⅱ. 5** Figure. S5. TonEBP diffuses along DNA

**Ⅱ. 6** Figure. S6. TonEBP depletion induces R-loop-dependent replication stress

**Ⅱ. 7** Figrue. S7. UV induced m6A RNA methylation is mainly focused on chromatin and m6A co-localizes with R-loop

**Ⅱ. 8.** Figure. S8. Figure S8. m6A RNA methylation by METTL3 is necessary for RNaseH1 recruitment

**Ⅱ. 9.** Figure S9. TonEBP-mediated pol k recruitment depends on METTL3

**Ⅱ. 10.** Figure. S10. Interaction of TonEBP with METTL3 is required for DNA damage-induced cell survival

**Ⅱ. 11** Figure. S11. Interaction of METTL3 with TonEBP is required for DNA damage-induced cell survival

**Ⅱ. 12.** Figure. S12. TonEBP binds with RNaseH1

**Ⅱ. 13.** Figure. S13. Blockade of R-loop formation reduces m6A induction on DNA damage sites

**Ⅱ. 14.** Figure. S14. Recruitment of YTHDF2 to R-loops is dependent on TonEBP

**Ⅱ. 15.** Figure. S15. S9.6 immunostaining without nucleolar structures

**Ⅱ. 16.** Figure. S16. There is no PLA signal with single antibody

**Ⅲ. Supplementary tables**

**Ⅲ. 1.** Table S1. Materials

**Ⅲ. 2.** Table S2. List of oligomers

**Ⅰ. Supplementary methods**

**Ⅰ.1.** **Tandem affinity purification (TAP) and mass spectrometry analysis**

TAP system and mass spectrometry were performed as described previously (1). HEK293 cells, transfected with Flag-tagged TonEBP plasmid, were lysed and mixed with anti-Flag M2 resin (Sigma-Aldrich). After 4 hrs, Flag-tagged TonEBP and bound proteins were eluted with 3x Flag peptide (Sigma-Aldrich). The molecular identities of the eluted peptides were detected by mass spectrometry (LC-MS/MS, program: Scaffold).

**Ⅰ.2.** **Single-molecule DNA curtain assay**

*Lambda DNA preparation*

Lambda phage DNA (λ-DNA) was purchased from NEB. For the DNA curtain assays, 15 nM λ-DNA was mixed with 1 μM oligomers containing COS sequences in T4 DNA ligase buffer (NEB) (Supplementary Table 2). For single-tether DNA curtain assay, Bio_COS_L/COS_R or Bio_COS_R/COS_L were used, while Bio_COS_R/Dig_COS_L or Bio_COS_L/Dig_COS_R were used for double-tether DNA curtain. The mixture was heated at 65 °C for 10 min and then cooled down to 23°C for annealing. Oligomers were ligated to λ-DNA by adding 5 units of T4 DNA ligase (NEB). After the ligases were heat-inactivated, excessive oligomers were removed by S-400 spin column (Illusta MicroSpin^TM^ S-400, GE Healthcare).

For λ-DNA with a single R-loop, a specially-engineered λ-DNA, λ-I3, was used, which contains seven nickase (Nt.BspQI) sites (2). The λ-I3 was treated with Nt.BspQI (0.6 units/ul) at 50°C for 1 hr. The nickase was degraded by proteinase K at 50°C. Then 400 times excessive Lambda_R-loop oligomers along with oligomers containing COS sequences were mixed with the nickase-treated λ-I3 T4 DNA ligase buffer (Supplementary Table 2). The mixture was heated to 70°C to heat-inactivate proteinase K and then slowly cooled down to 23°C. All oligomers were ligated by T4 DNA ligase, which then was inactivated 65°C. The λ-I3 containing a bubble was filtered by S-400 spin column (Illusta MicroSpin^TM^ S-400, GE Healthcare). Then 5 nM of the bubble-containing λ-I3 was mixed and annealed with 2 uM Lambda_R-loop RNA, which was complementary to the bubble sequence.

Another λ-DNA containing a different R-loop construct was prepared by using restriction enzyme digestion and ligation. Biotinylated λ-DNA as described above was digested by PspXI (NEB). A new R-loop construct, which has phosphorylated single-stranded overhangs complementary to PspXI cohesive end but does not have PspXI recognition sequence, was prepared by annealing Lambda R-loop2, Lambda R-loop2_comp, and Cy5-labeled R-loop RNA at equimolar ratio (Supplementary Table S2 and Supplementary Figure S4E). The PspXI-cleaved λ-DNA and the new R-loop construct were mixed at 1:200 molar ratio and ligated with T4 DNA ligase (NEB). Excessive RNA was removed by S-400 spin column (Illustra MicroSpin^TM^ S-400, GE Healthcare).

*Total internal reflection fluorescence microscope (TIRFM)*

Prism-type total internal reflection fluorescence microscope (TIRFM) was custom-built with an inverted fluorescence microscope (Eclipse Ti-2, Nikon). The solid-state 488 nm and 637 nm lasers (200 mW, OBIS, Coherent Laser) were used to excite quantum dot (Qdot) and Cy5, respectively. The laser beam passed through a dove prism and was totally reflected at the boundary between a fused-silica slide and buffer with a high incident angle. The fluorescence from Qdots or Cy5 was collected by 60x water-immersion objective lens (CFI Plan Apo VC 60X, Nikon) and imaged on each of two EM-CCD cameras (iXon 897, Andor Technology). Long pass filter (ET500lp for Qdot or ET650lp for Cy5, Chroma Technology) was used to block the 488 nm or 637 nm laser light. The data were collected by the NIS-Element software (Nikon).

*Flowcell preparation for the DNA curtain assay*

The flowcell for DNA curtain assay was prepared following the previous protocol (3,4). Fused-silica slides with two holes were cleaned by successive treatment with 2% Hellmanex III (Sigma), acetone, 1 M sodium hydroxide, and DI water. The slides were dried with N_2_ gas for the nano-patterning. The narrow and wide chromium nano-barriers were fabricated on the cleaned slide by the following nano-patterning processes: the spin-coating of e-beam resistor (PMMA), electron beam lithography, development, chromium deposition by electron beam evaporator, and lift-off. Then the flowcell with microchannel was constructed by gluing the patterned slide and a coverslip with a double-sided tape. Nanoports (IDEX) were attached on the holes to connect the flowcell with a fluidic system. The flowcell was cleaned with DI and lipid buffer (20 mM Tris-HCl pH 8.0 and 100 mM NaCl). All lipids were purchased from Avanti-Polar Lipids. Liposomes consisting of DOPC (1,2-dioleoyl-*sn*-glycero-phosphocholine), 0.5% biotinylated-DPPE (1,2-dipalmitoyl-*sn*-glycero-3-phosphoethanolamine-N-(cap biotinyl)), and 8% mPEG 2000-DOPE (1,2-dioleoyl-*sn*-glycero-3-phosphoethanolamine-N-[methoxy(polyethylene glycol)-2000]) were deposited on the microchannel surface. The lipid bilayer was promoted on the surface by 20 min incubation after free liposomes were washed with lipid buffer. Only for double-tether DNA curtain, 0.1 mg/ml of anti-digoxigenin (11214667001, Roche) in lipid buffer was added to the flowcell and nonspecifically adsorbed on the wide chromium nano-barriers. For single-tether DNA curtain, this step was skipped. BSA buffer (40 mM Tris-HCl pH 8.0, 50 mM NaCl, 2 mM MgCl_2_, and 0.4% BSA) was then injected to further passivate the surface that was not covered with lipid bilayer. The biotinylated λ-DNA was attached to biotinylated lipid via streptavidin. The flowcell was connected with a fluidic system consisting of a syringe pump and a 6-way injection valve and then placed on the TIRFM. Under the continuous buffer flow, DNA molecules were moving along the flow. At the first barrier, the biotinylated end was stuck, and DNA molecules were stretched. In the double-tether DNA curtain, the other end of λ-DNA was tagged with digoxigenin, which was tethered to the wide barrier coated with anti-digoxigenin and hence DNA molecules were double-tethered between two barriers and held stretched even in the absence of continuous flow.

*R-loop binding of Yc1 in DNA curtain*

To examine the binding of Yc1 to R-loop in equilibrium, single-tether DNA curtain was used. 5 nM R-loop containing λ-DNA was incubated with 35 nM Yc1 in TonEBP buffer with 50 mM NaCl (total volume: 14 μL) at 23 °C for 2 hrs. The reactant was then 10 times diluted in imaging buffer (TonEBP buffer with 50 mM NaCl, 1.6% glucose, and 0.1x gloxy) and injected into the flowcell, followed by 10 min incubation for anchoring λ-DNA onto the lipid bilayer. During the incubation steps, external light was blocked to prevent the photobleaching of Cy5 on RNA. Firstly, R-loop formation in the DNA curtain was imaged at 0.5 ml/min under 637 nm laser illumination. Then Yc1, which had 3xFLAG at N-terminus, was labeled with ~3 nM FLAG-antibody conjugated Qdot (SiteClick Qdot 565 antibody labeling kit, S10450, Invitrogen) in the flowcell and imaged with 488 nm laser excitation. The mechanism by which Yc1 identifies R-loop was investigated using double-tether DNA curtain. 100 nM Yc1 was pre-incubated with FLAG-antibody conjugated Qdot on ice for 15 min. After DNA curtains were formed with R-loop containing λ-DNA, 5 nM of Qdot-conjugated Yc1 was injected. When the maximum concentration of Yc1 arrived at the curtains, the flow was turned off and images were taken. All image data were collected with 10 Hz of frame rate.

*Diffusion of Yc1 in DNA curtain*

To investigate the diffusive motion of Yc1, we used double-tether DNA curtain with λ-DNA (NEB) that was labeled with biotin and digoxigenin at either end without R-loop. We pre-incubated 5 nM λ-DNA with 35 nM Yc1 in in TonEBP buffer with different NaCl concentrations (total volume: 14 μL) at 23°C for 2 hrs. The Yc1 was labeled with ~3 nM FLAG-antibody conjugated Qdot (SiteClick Qdot 565 antibody labeling kit, S10450, Invitrogen) in the flowcell. The motion of Yc1 was imaged in imaging buffer (TonEBP buffer with 50 mM NaCl, 1.6% glucose, and 0.1x gloxy) without buffer flow. All image data were collected with 10 Hz of frame rate.

*DNA curtain data analysis*

All images collected by NIS-Element were converted into 8-bit TIFF format. All analyses were performed by ImageJ (NIH). The particle tracking was carried out by the ImageJ plug-in, MOSAIC suit particle tracker. For the one-dimensional (1D) diffusion coefficient, at least more than 300 frames were tracked. By Matlab (Mathworks), the 1D diffusion coefficient (*D*) of each Yc1 molecule was calculated from mean square displacement (*MSD*), which is mathematically given as

$$MSD \left( n, N \right)= \sum_{i=1}^{N-n} \frac{{(Y_{i+n}-Y_{i})}^{2}}{N-n}=2Dn\Delta t$$

where *N* is the total number of frames, *n* is the measurement window ranging from 1 to *N*, *Δt* is the time interval between frames, and *Y* is the position of Yc1 along DNA. The standard deviation (*SD*) in *MSD* was used for error, which was obtained from the equation,

$$SD=\sqrt{\frac{\left( 2Di\Delta t \right)^{2}(2t^{2}+1)}{3i(N-i+1)}}$$

*D* was obtained from the linear fitting of *MSD* with the first five data points because the error of *MSD* becomes large as the frame increases (Supplementary Figure S5).

The diffusion limit of rotational motion was calculated based on modified Schurr’s model, the total friction is given like

$\xi_{trans}^{total}=6\pi\eta R+\left( \frac{2\pi}{10 BP} \right)^{2}\left[ 8\pi\eta R^{3}+6\pi\eta R{(R_{OC})}^{2} \right]$ (5).

Here, *η* is the viscosity of water (0.9 mPa sec), *BP* is the length of 1 bp of duplex DNA (0.34 nm), *R* is the radius of protein, and *R_OC_* is the separation between DNA axis and the center of mass of protein.

The theoretical limit of diffusion coefficient for the rotational motion around helix is given from Einstein equation,

$D=\frac{k_{B}T}{\xi_{trans}^{total}}$.

In the DNA curtain assay, Yc1 was labeled with Qdot, and hence *R_OC_* is given as the sum of TonEBP radius and the hydrodynamic radius of antibody-conjugated Qdot. The radius of Yc1 is ~ 4 nm from its x-ray crystal structure (PDB ID: 1imh) (6). The hydrodynamic radius of antibody-conjugated Qdot is ~ 13 nm based on the information of maker (Invitrogen). So, *R_OC_* is approximately 17 nm. *k_B_T* is approximated to 4.1 pN nm at 23°C. Finally, the rotational limit of diffusion coefficient is ~ 0.057 μm^2^/sec, which is smaller than the diffusion coefficients for Yc1 at all measured NaCl concentrations, suggesting that Yc1 slides along the DNA backbone without rotation around the helix.

**Ⅰ.3.** **Immunofluorescence assay for R-loop without nucleolus**

To remove nucleolar structures of U2OS cells, the cells were trypsinized, and then pre-warmed 75 mM KCl was mixed with cells at 37°C for 12 min. Cells were then fixed with cold fixative solution (methanol/acetic acid 3:1). After fixation step, the cell suspension was spread onto a clean slide and exposed to 90°C steam for 60 s. Cells were incubated with blocking buffer (3% bovine serum albumin (BSA) and 0.5% Triton X-100 in 1xPBS) for 1 h and then incubated with S9.6 antibody (1:1000) in blocking buffer overnight at 4°C. Cells were washed with washing buffer (1xPBS supplemented with 0.1% Triton X-100), followed by incubation with mouse AlexaFluor 499-conjugated antibody (1:500) in wash buffer for 1 h at room temperature. After washing, cells were mounted using ProLong® Gold antifade reagent (Vector Laboratories). Confocal images were acquired with an LSM70 confocal microscope (Carl Zeiss). Image acquisition and analysis were performed with ZEN2.1 software.

**Ⅰ.4.** **References**

1. Kang, H.J., Park, H., Yoo, E.J., Lee, J.H., Choi, S.Y., Lee-Kwon, W., Lee, K.Y., Hur, J.H., Seo, J.K., Ra, J.S. *et al.* (2019) TonEBP Regulates PCNA Polyubiquitination in Response to DNA Damage through Interaction with SHPRH and USP1. *iScience*, **19**, 177-190.

2. Cheon, N.Y., Kim, H.S., Yeo, J.E., Scharer, O.D. and Lee, J.Y. (2019) Single-molecule visualization reveals the damage search mechanism for the human NER protein XPC-RAD23B. *Nucleic acids research*, **47**, 8337-8347.

3. Lee, J.Y., Finkelstein, I.J., Arciszewska, L.K., Sherratt, D.J. and Greene, E.C. (2014) Single-Molecule Imaging of FtsK Translocation Reveals Mechanistic Features of Protein-Protein Collisions on DNA. *Mol Cell*, **54**, 832-843.

4. Lee, J.Y. and Greene, E.C. (2011) Assembly of recombinant nucleosomes on nanofabricated DNA curtains for single-molecule imaging. *Methods in molecular biology (Clifton, N.J.)*, **778**, 243-258.

5. Bagchi, B., Blainey, P.C. and Xie, X.S. (2008) Diffusion constant of a nonspecifically bound protein undergoing curvilinear motion along DNA. *J Phys Chem B*, **112**, 6282-6284.

6. Stroud, J.C., Lopez-Rodriguez, C., Rao, A. and Chen, L. (2002) Structure of a TonEBP-DNA complex reveals DNA encircled by a transcription factor. *Nat Struct Biol*, **9**, 90-94.

7. Lin, S., Choe, J., Du, P., Triboulet, R. and Gregory, Richard I. (2016) The m6A Methyltransferase METTL3 Promotes Translation in Human Cancer Cells. *Molecular Cell*, **62**, 335-345.

**Ⅱ. Supplementary Figures**

**
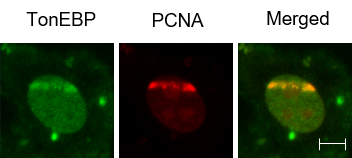
**

**Figure S1.** **TonEBP co-localizes with PCNA in DNA damage sites.** U2OS cells were subjected to laser microirradiation and immunostained for TonEBP and PCNA. Representative images of a nucleus were presented. Scale bar stands for 2 μm.

**
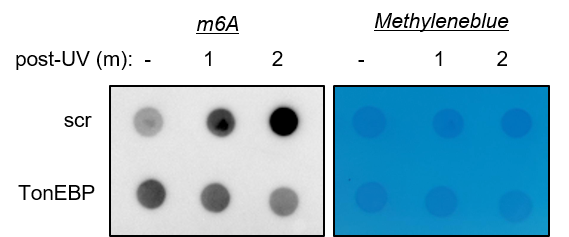
**

**Figure S2. TonEBP induces UV-induced m6A RNA methylation.** U2OS cells were transfected with scrambled siRNA (scr) or TonEBP-targeting siRNA (TonEBP) for 24 hrs. Cells were subjected to UV irradiation and poly A (+) mRNA was isolated and dot immunoblotted for m6A.

**
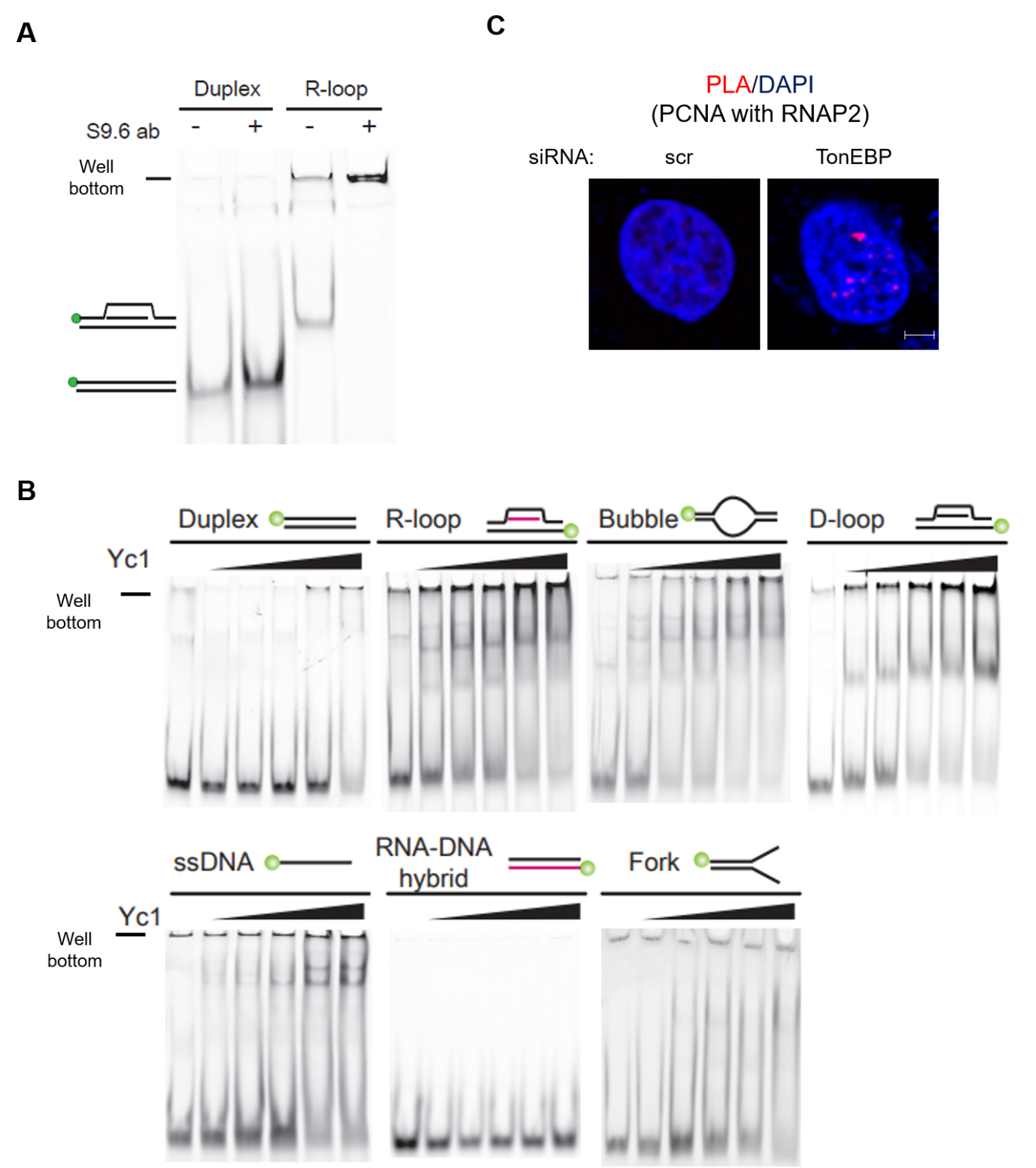
**

**Figure S3. TonEBP preferentially binds R-loop, especially the displaced ssDNA *in vitro***

(A) Electrophoretic mobility shift assay (EMSA) of Cy3-labeled duplex DNA and R-loop. Positions of duplex DNA and R-loop are shown at left. Where indicated, S9.6 antibody was added. S9.6 bound probe is found at the bottom of wells. (B) Gel images for EMSA of Yc1 with different types of DNA substrates such as duplex, R-loop, bubble, D-loop, ssDNA, RNA-DNA hybrid, and fork (Y-shape) (Supplementary Table S2). 30 nM DNA substrate was incubated with Yc1 at 0 nM, 12.5 nM, 25 nM, 50 nM, 100 nM, 200 nM, and 400 nM from left to right. (C) U2OS cells were transfected with scrambled or TonEBP siRNA. PLA was performed with anti-PCNA and RNAP2 antibodies to assess transcription-replication collision. Scale bar stands for 2 μm.

**
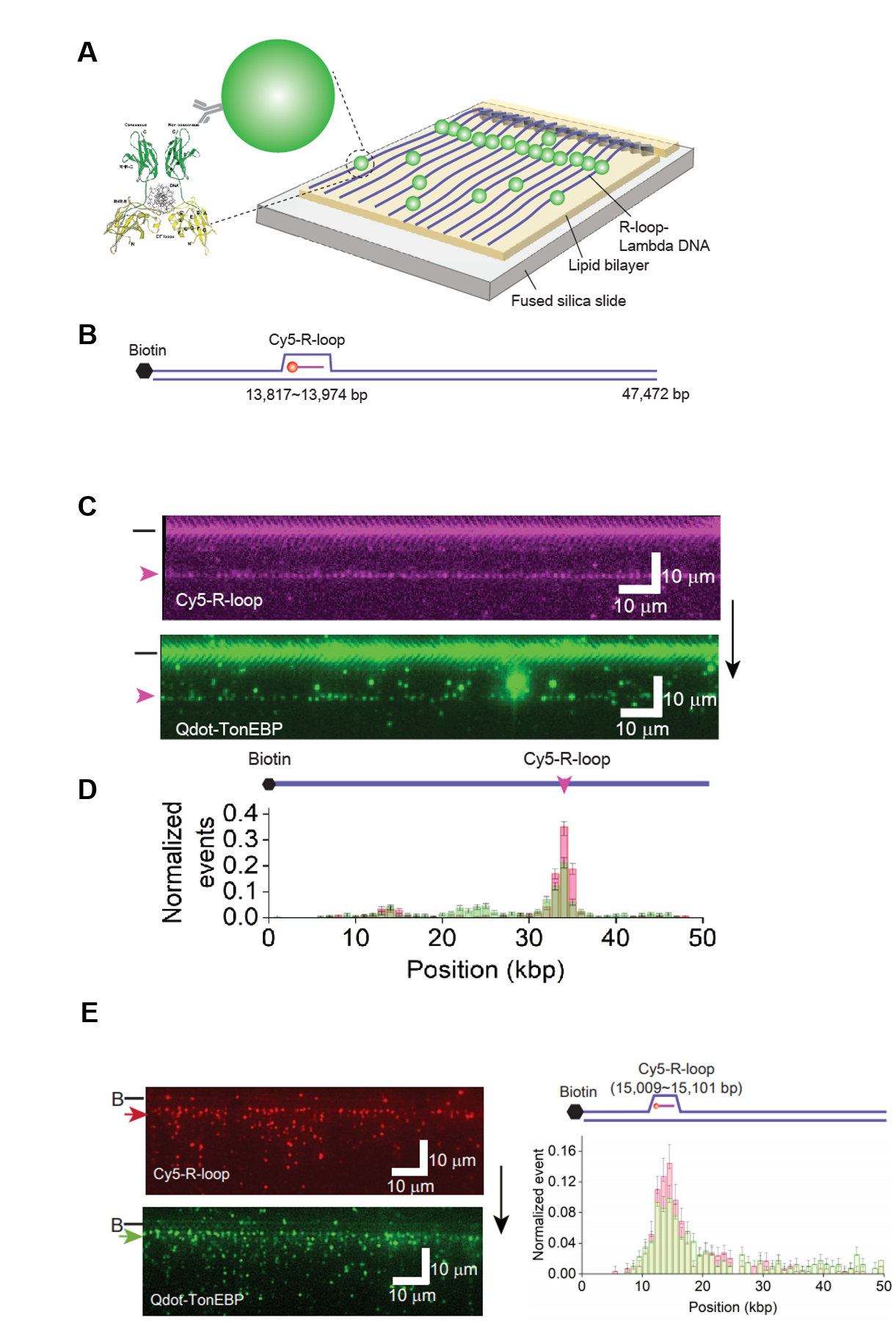
**

**Figure S4. Single-molecule visualization of TonEBP and R-loop interaction *in vitro*** (A) Schematic of single-tether DNA curtain, which was formed by lambda phage (λ) DNA molecules that were modified with biotin only at one end. Yc1 (PDB ID: 1imh) was conjugated with Anti-FLAG Qdot. (B) Construct of biotinylated λ-DNA containing R-loop, which located between 13,817 bp and 13,974 bp and was tagged with Cy5. (C) Two-color images of single-tether DNA curtain. Here the biotin end was reversed, so biotin was labeled at the opposite end to Figure 3C and D. (D) Binding distribution of Cy5-R-loop (magenta) and Qdot-Yc1 (green) for the reversed λ-DNA. Both peaks were well-overlapped at the R-loop location. (E) Preferential binding of Yc1 to another R-loop construct which is placed at different location of λ-DNA. The new R-loop construct was inserted into λ-DNA by restriction enzyme (PspXI) digestion and ligation. (Left) DNA curtain images for Cy5-R-loop (top) and Qdot-TonEBP (bottom). Black bar and magenta arrow left to each image represent barrier and R-loop, respectively. (Right) Binding distribution of Cy5 and Qdot, both of which overlap well at the R-loop position (numbers of molecules for Cy5 and Qdot are 369 and 291, respectively). The construct of lambda DNA containing R-loop, which is different from that in Figure S4B, is shown above the distribution histogram.

**
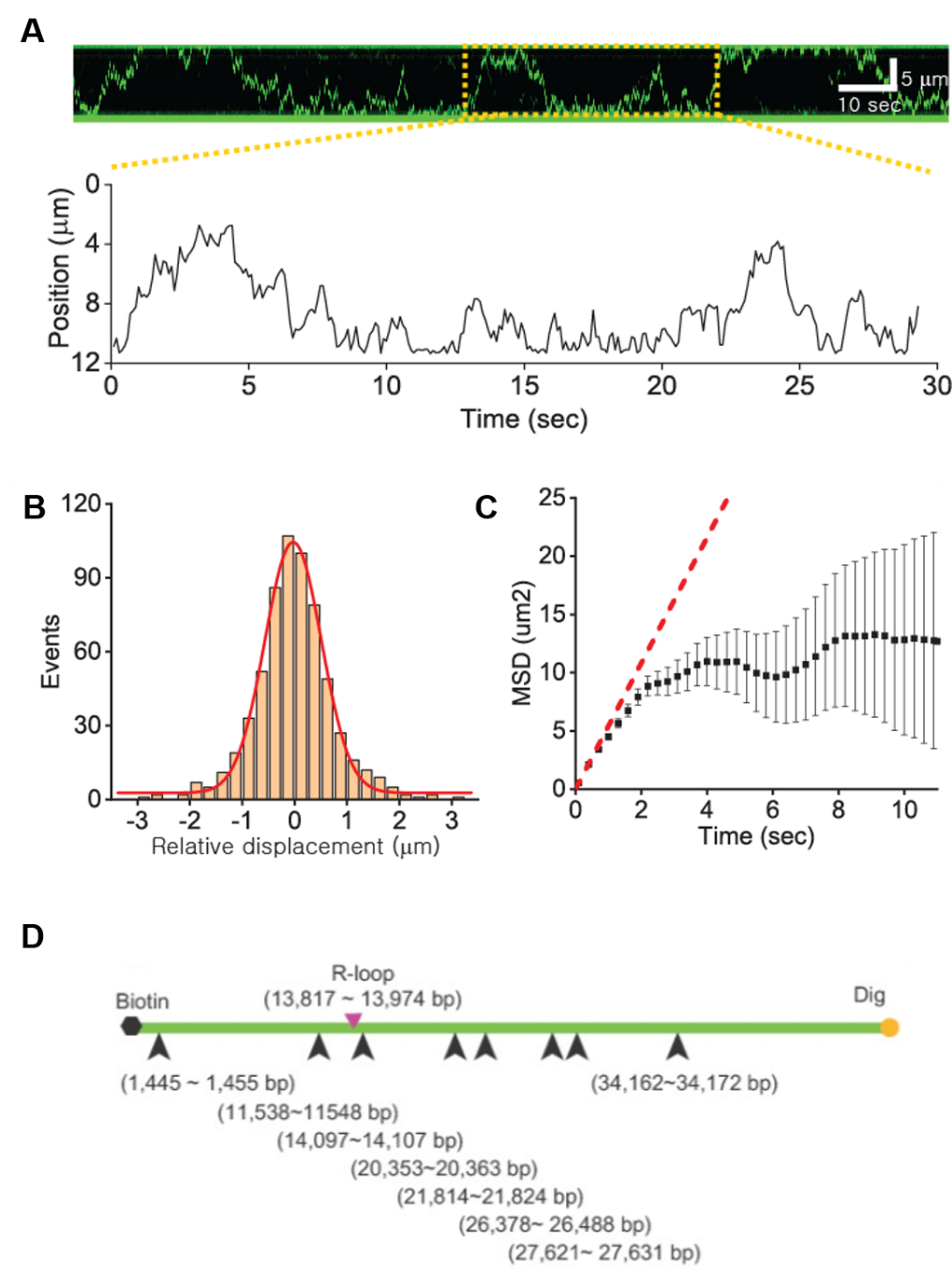
**

**Figure S5. TonEBP diffuses along DNA**

(A) (Top) Kymograph showing diffusion of Yc1 through λ-DNA without R-loop and (bottom) particle-tracking trace of the particle from the yellow dashed box region. (B) Histogram for relative displacement from the particle-tracking data in (A). The histogram was fitted by Gaussian function with the center zero (red line) indicating that the movement of Yc1 was Brownian motion. (C) Mean square displacement (MSD) of tracked particle in (A). The diffusion coefficient in different salt concentrations (shown in Fig. 3G) was calculated from linear fitting (red dashed line) of the first 5 data points of this MSD. (D) Locations of TonE (black arrow heads) on λ-DNA containing R-loop (magenta triangle). Wild-type λ-DNA without R-loop has one more TonEBP binding sequence at the R-loop position.

**
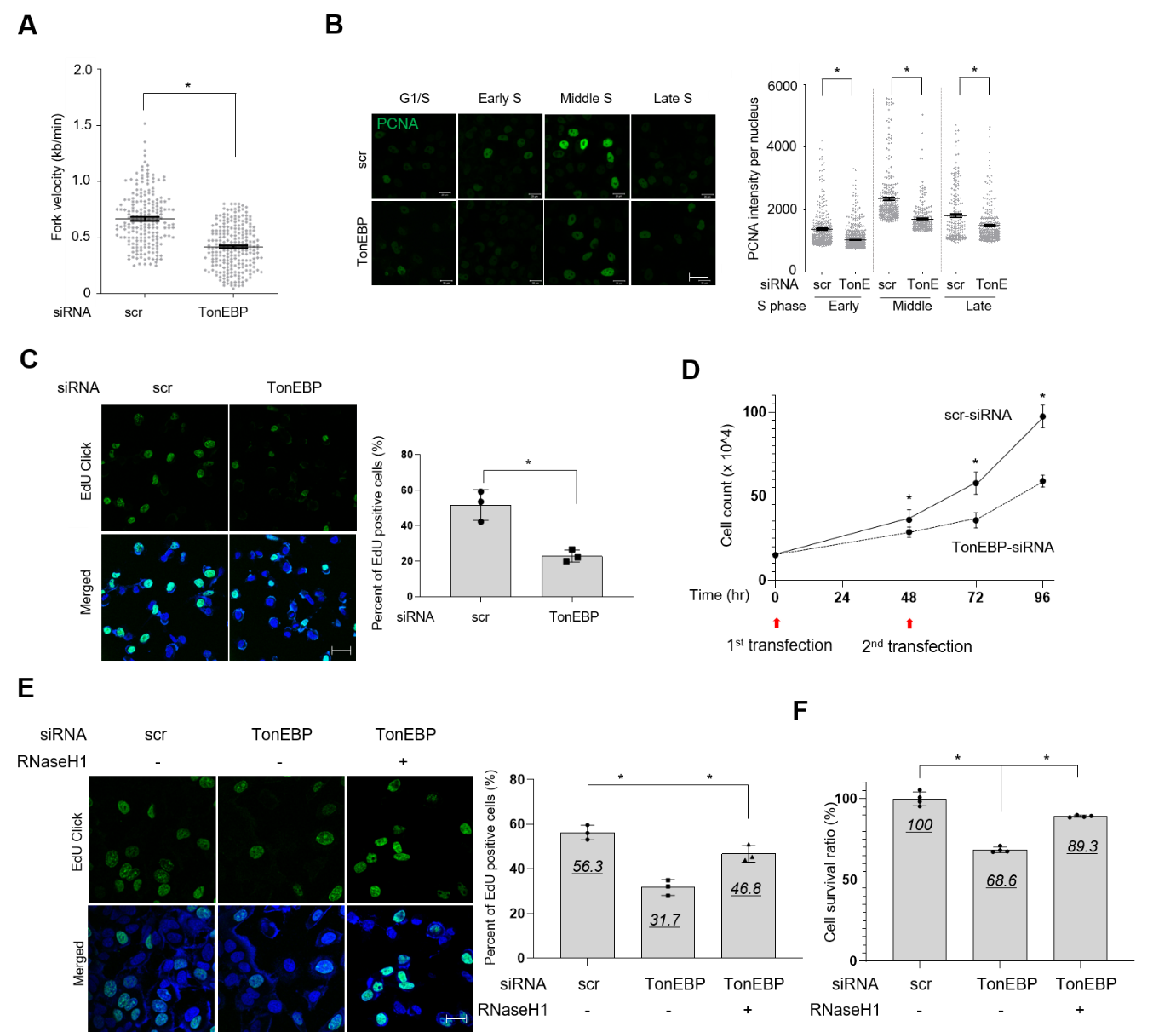
**

**Figure S6. TonEBP depletion induces R-loop-dependent replication stress**

(A) Analysis of replication fork velocity using DNA molecular combing assay. The iodo-deoxyuridine (IdU)-labeled track over chloro-deoxyuridine (CldU)-labeled track was used to assess fork velocity in > 300 well-isolated DNA fibers from three independent experiments. Mean ± SD, *p < 0.01. (B) U2OS cells treated with aphidicolin (10 μg/ml) for 16 hrs. Chromatin bound PCNA was immunostained 0 hr (G1/S), 3 hrs (early S), 6 hrs (middle S), and 9 hrs (late S) after removal of aphidicolin. Representative images are on the left. PCNA intensity in each condition was measured from > 200 cells obtained from 3 independent experiments as shown on the right. Mean ± SD, *p < 0.01. (C) U2OS cells were transfected with siRNA as indicated. Cells were then subjected to EdU-Click labeling analysis. Left: representative images of cells in each condition. Right: percent of EdU positive cells were measured. Mean ± SD, n = 3. *p < 0.01. Scale bar stands for 20 μm. (D) U2OS cells were transfected twice with the same siRNA at 0 and 48 hrs as indicated. Live cells were counted at 48, 72, and 96 hrs. Mean ± SD, n = 3. *p < 0.01. (E) siRNA transfected cells were transfected a second time with a plasmid expressing RNaseH1 as indicated. EdU-Click labeling analysis was performed and EdU labeling was visualized. Left: representative images. Right: percent of EdU positive cells was measured from > 30 cells. Mean ± SD, n = 3, *p < 0.01. Scale bar stands for 10 μm. (F) Cells were transfected as in (E). Live cells were counted 24 hrs later.

**
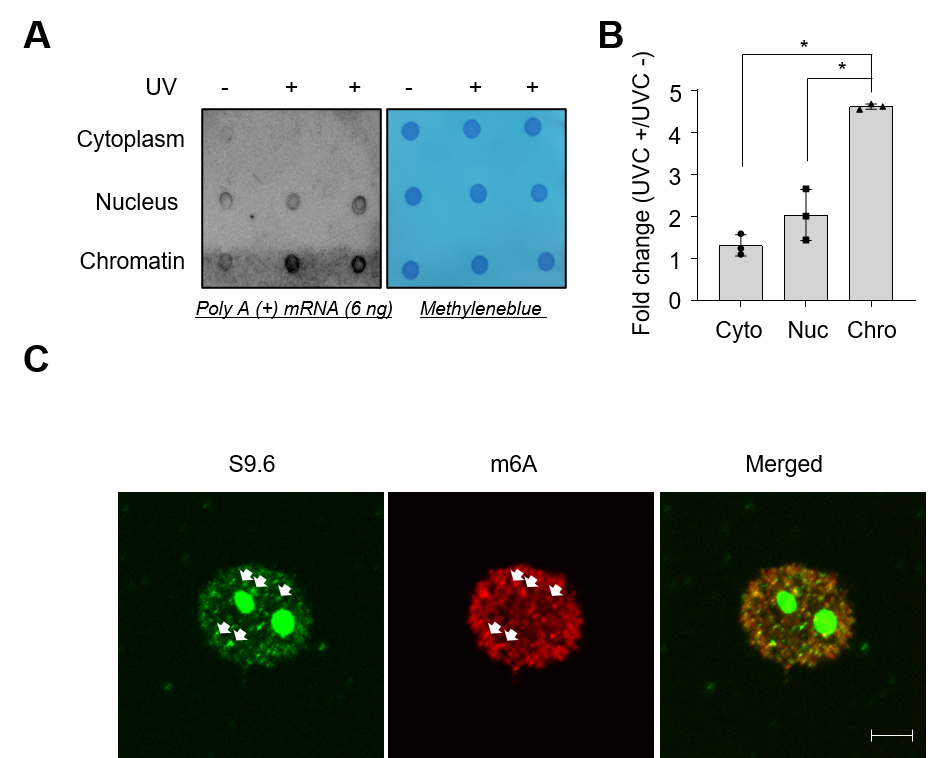
**

**Figure S7. UV induced m6A RNA methylation is mainly focused on chromatin and m6A co-localizes with R-loop.** (A-B) U2OS cells were subjected to 60 J/m^2^ UVC irradiation and fractionated from cytoplasm, nucleus plasma to chromatin-bound. (A) Left: poly A (+) mRNA from cells was subjected to dot blot analysis with anti-m6A antibody. Right: loading control stained with methylene blue. (B) Fold changes between UVC + and UVC - were measured from three independent experiments; Mean ± SD, *p < 0.01. (C) U2OS cells were fixed and immunostained for S9.6 and m6A (white arrows), which overlapped. Scale bar stands for 2 μm.

**
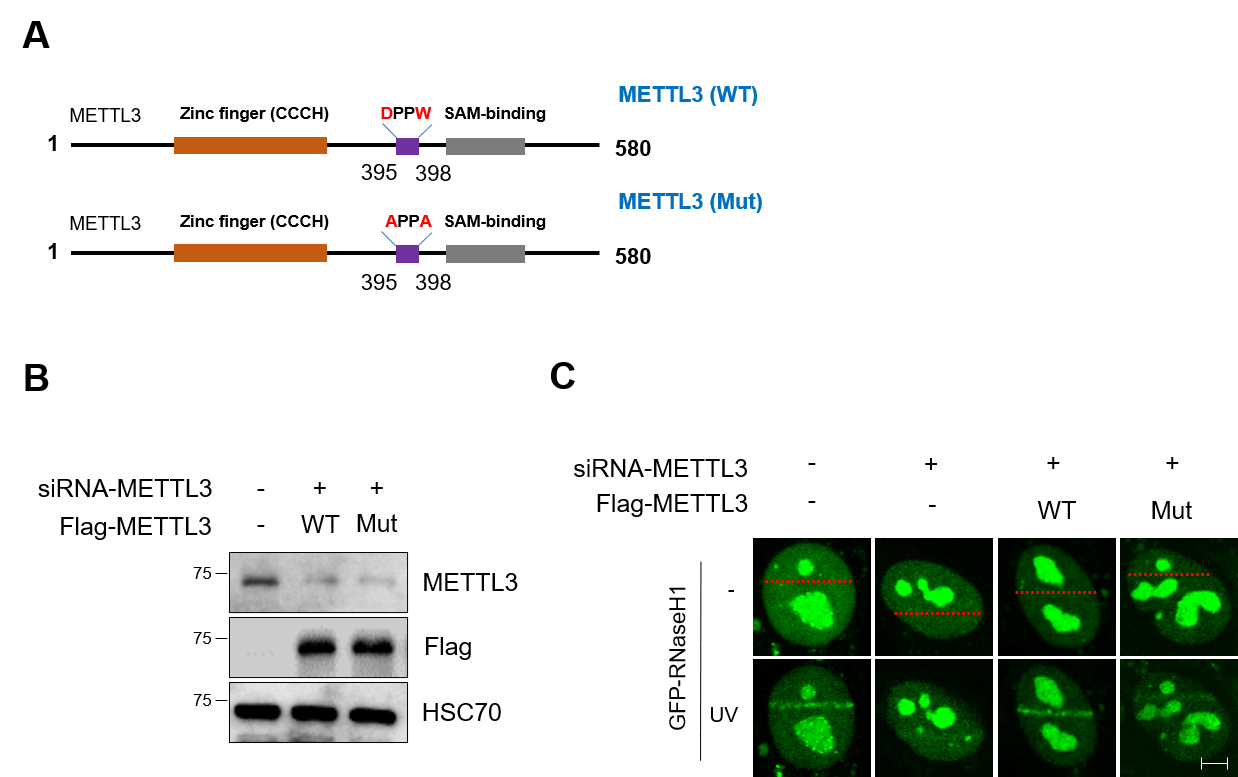
**

**Figure S8. m6A RNA methylation by METTL3 is necessary for RNaseH1 recruitment**

(A) Schematic diagram for catalytically active (top) and inactive METTL3 (bottom), in which D395 and W398 were replaced by A (7). (B) Immunoblot assay for METTL3 knockdown (top), over-expression of catalytically active and inactive METTL3 for complementation (middle) and loading control with heat shock cognate 71 kDa protein (HSC70) (bottom). (C) RNaseH1 recruitment on laser-microirradiated sites in U2OS cells containing catalytically active (WT) or catalytically inactive (Mut) METTL3. Microirradiated region was denoted with red dashed line. Scale bar stands for 2 μm.

**
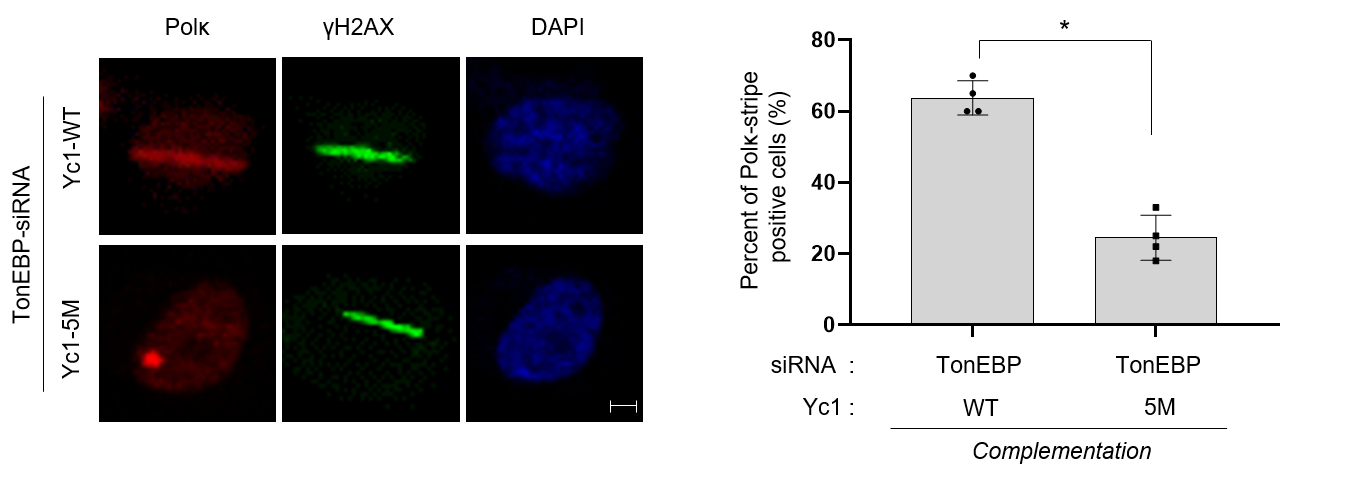
**

**Figure S9. TonEBP-mediated pol κ recruitment depends on METTL3**

TonEBP in U2OS cells was depleted by siRNA. The cells were also transfected with plasmids expressing wild-type (WT) Yc1 (left upper panel) or 5M (left lower panel) and then subjected to laser-microirradiation followed by incubation for 2 min and immunostained for Pol κ and γH2AX. Left: representative images. Right: percentage of Pol κ laser stripe-positive cells from 10 cells. Mean ± SD, n = 4. *p < 0.01. Scale bar stands for 1 μm.

**
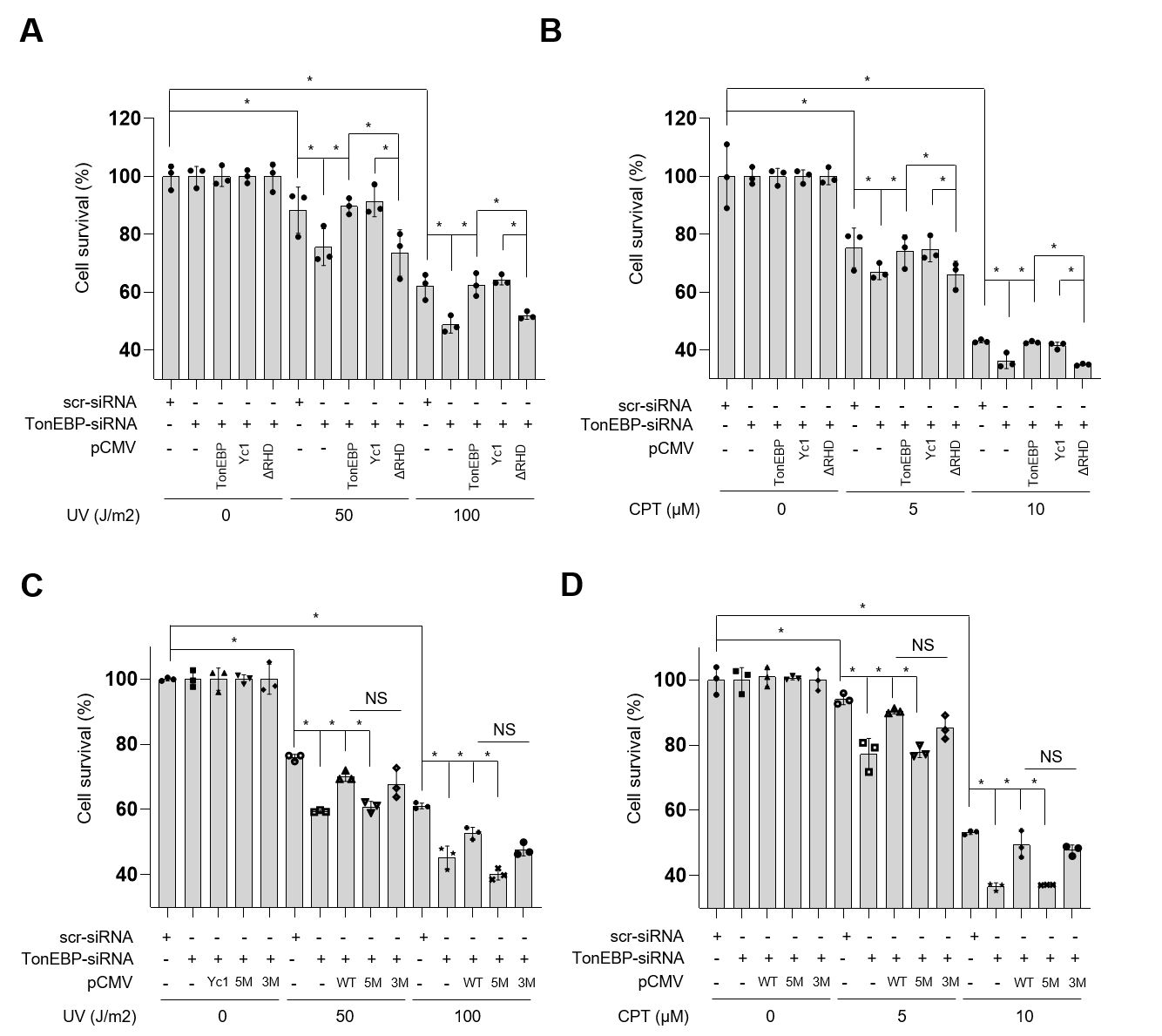
**

**Figure S10. Interaction of TonEBP with METTL3 is required for DNA damage-induced cell survival**

(A) U2OS cells were transfected with various combinations of siRNAs as indicated. The cells were then transfected a second time with pCMV expressing full length TonEBP, Yc1, or ΔRHD. Cell survival was measured after treatment with 0 to 100 J/m^2^ of UV. Mean ± SD, n = 3. *p < 0.01. (B) Cells were transfected as in (A). Cell survival was measured after treatment with 0 to 10 μM CPT. (C) Cells transfected with various combination of siRNAs were transfected a second time with pCMV expressing Yc1, 5M, or 3M. Cell survival was measured after UV treatment. (D) Cells were transfected as in (C). Cell survival was measured after CPT treatment.

**
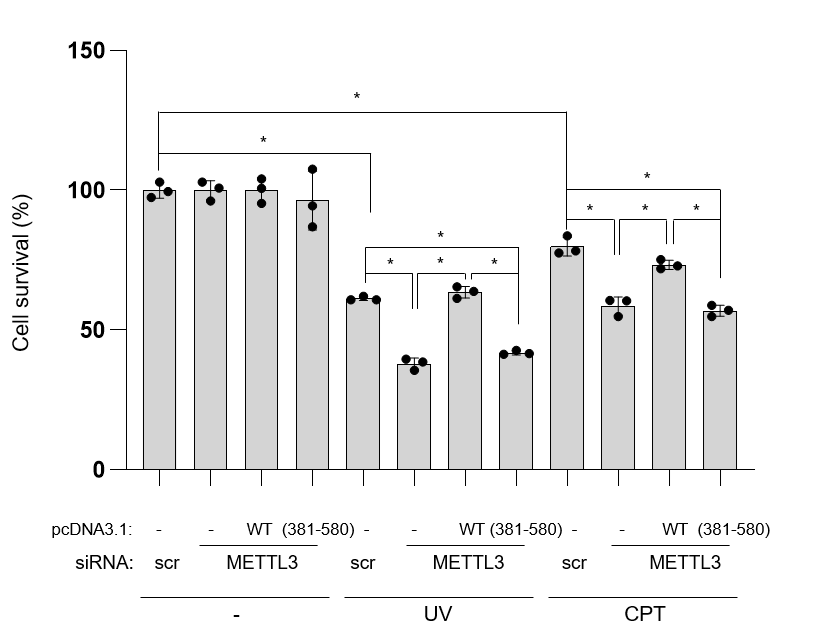
**

**Figure S11. Interaction of METTL3 with TonEBP is required for DNA damage-induced cell survival**

U2OS cells transfected with METTL3 siRNA were transfected second time with pcDNA3.1 expressing METTL3 (WT) or 381-580 as indicated. Cell survival was measured after treatment with UV or CPT.

**
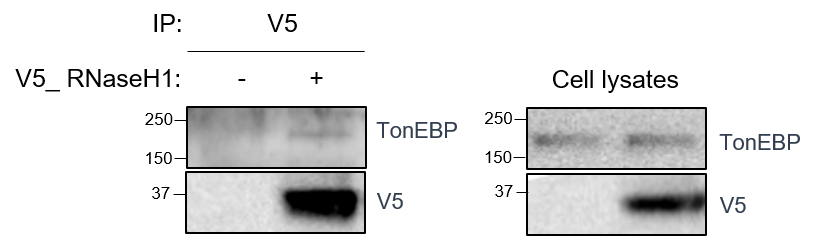
**

**Figure S12. TonEBP binds with RNaseH1**

U2OS cells were transfected with an empty plasmid or a plasmid expressing V5-RNaseH1. Cell lysates were immunoprecipitated with anti-V5 antibody. Precipitates and cell lysates were immunoblotted for TonEBP and V5.

**
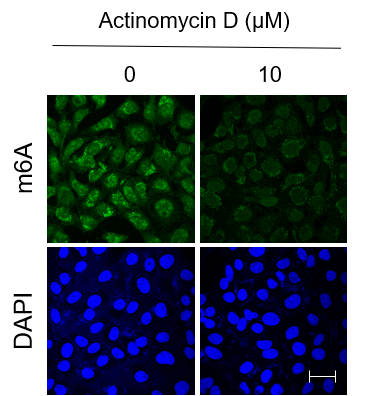
**

**Figure S13. Blockade of R-loop formation reduces m6A induction on DNA damage sites.** U2OS cells were pre-treated with 10 μM actinomycin D for 10 min. Cell were subjected to 60 J/m^2^ UVC light irradiation, fixed and immunostained for m6A RNA. Representative images of nuclei in each condition are shown. Scale bar stands for 20 μm.


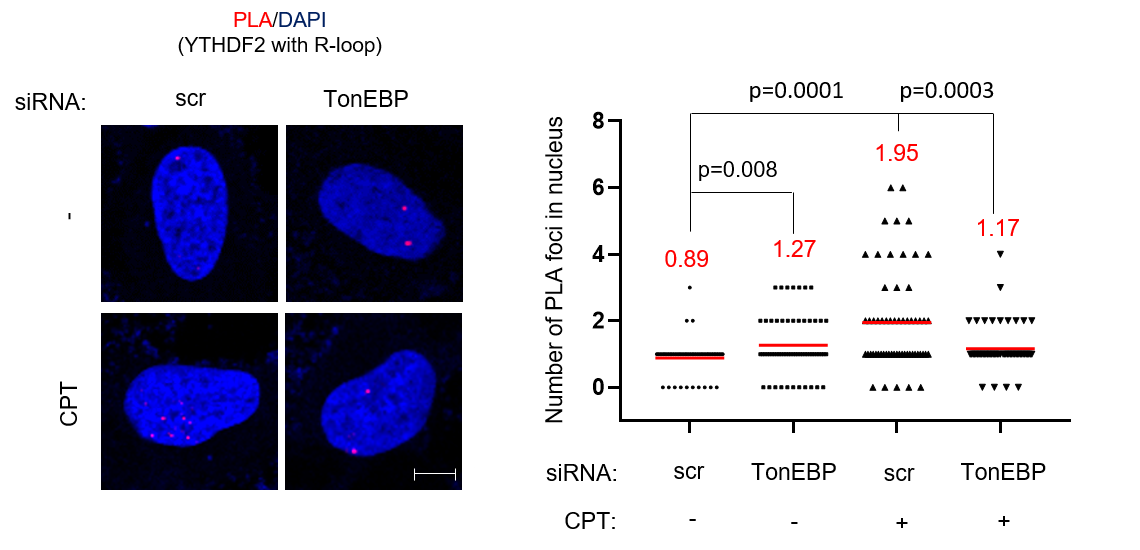


**Figure S14. Recruitment of YTHDF2 to R-loops is dependent on TonEBP**

siRNA-transfected cells were treated with 10 μM CPT followed by PLA for YTHDF2 and S9.6. Left: representative images. Right: the numbers of PLA dots per nucleus were counted in at least 60 nuclei from three independent experiments. Red line represents mean value, which is also denoted above in red. Scale bar stands for 2 μm.

**
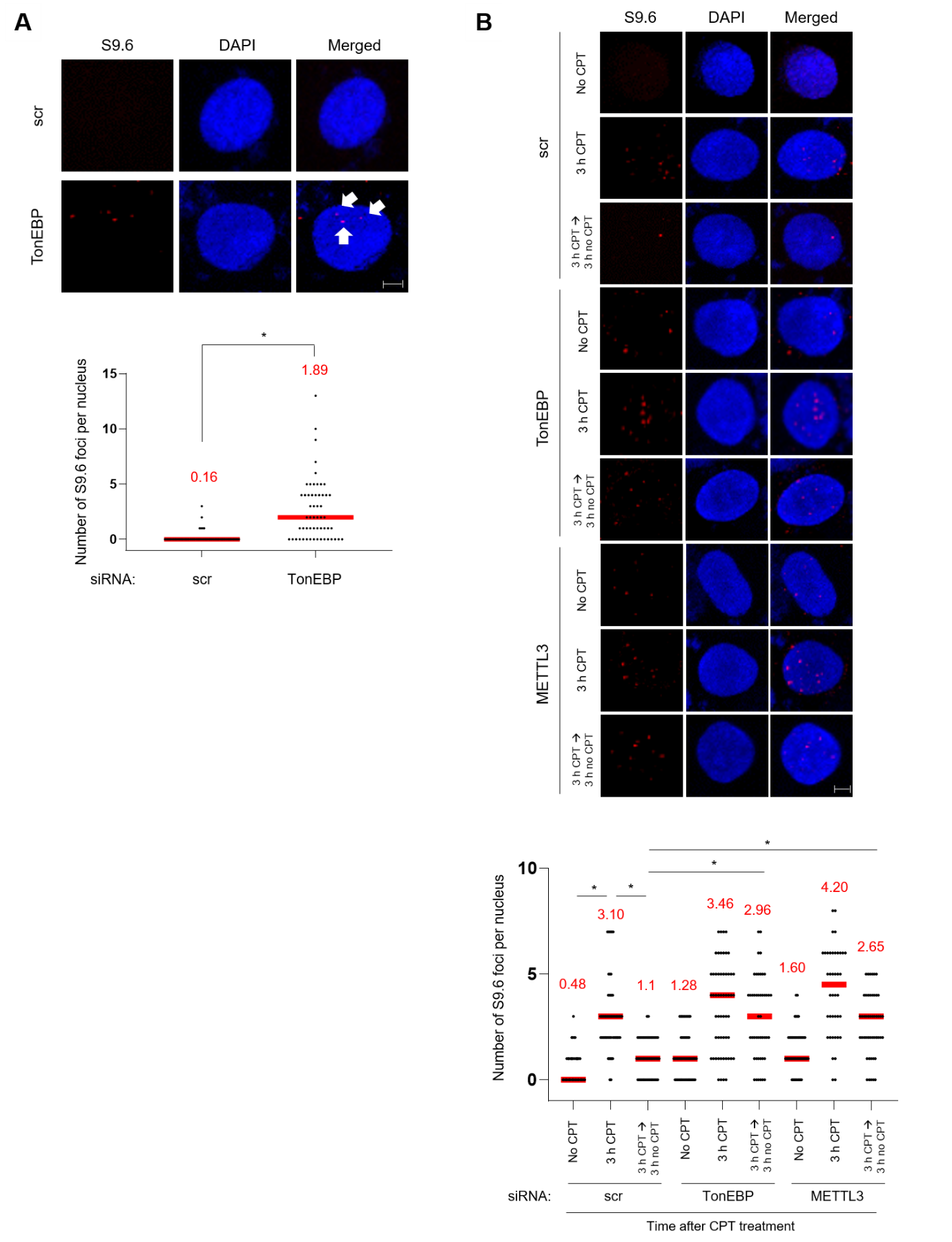
**

**Figure S15. S9.6 immunostaining without nucleolar structures**

**(A)** U2OS cells were transfected with scrambled siRNA (scr) or TonEBP-targeting siRNA (TonEBP) for 48 hrs. Nucleolin was eliminated after cells were fixed. Then the cells were immunostained for S9.6. (Top) Representative images, where nucleolin completely disappear. (Bottom) the S9.6 foci per nucleus were counted from 30 nuclei. Mean ± SD, *p < 0.01. (B) siRNA-transfected U2OS cells were either untreated (no CPT) or treated with 10 μM CPT for 3 hrs and analyzed immediately or after 3 hrs further incubation in fresh media without CPT. Then the cells were fixed, and nucleolin was removed, followed by S9.6 immunostaining. (Top) Representative images, where nucleolin completely disappear. (Bottom) the S9.6 foci per nucleus were counted from 90 nuclei. Mean ± SD, *p < 0.01. Scale bar stands for 1 μm.

**
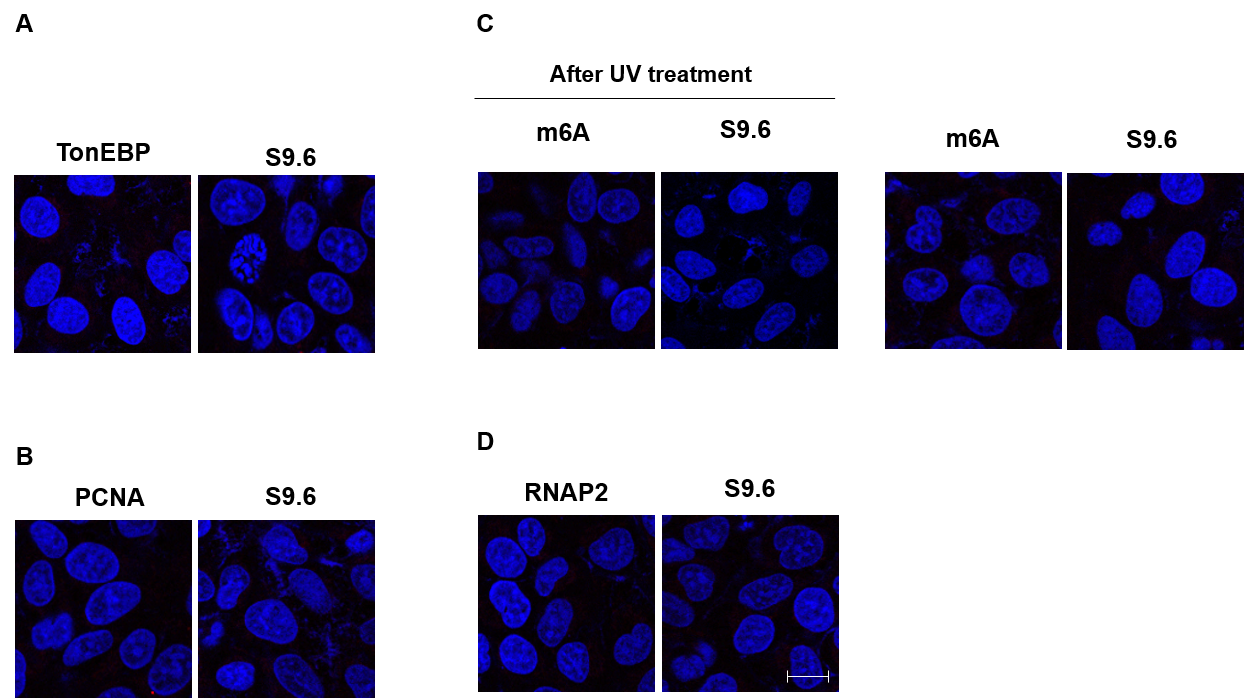
**

**Figure S16. There is no PLA signal with single antibody**

As a negative control, PLA experiments were carried out with single antibody of each target protein (A) TonEBP and S9.6, (B) PCNA and S9.6, (C) m6A and S9.6, and (D) RNA polymerase II (RNAP2) and S9.6 Scale bar stands for 5 μm.

**Ⅲ. Supplementary tables**

**Ⅲ. 1. Table S1. Materials**

| **REAGENT or RESOURCE** | **SOURCE** | **IDENTIFIER** |
| --- | --- | --- |
| **Antibodies** | | |
| TonEBP | Serum |  |
| METTL3 | ABclonal | A8370 |
| PCNA | Santa Cruz | sc-56 |
| phospho-H2A.X | Millipore | 05-636 |
| m6A | Synaptic systems | 202-003 |
| S9.6 | Santa Cruz | sc-56 HRP |
| Nucleolin | Cell signaling Technilogies | 14574 |
| RNAP2 | Bethyl | A300-653A |
| HSC70 | ROCKLAND | 200-301-A28 |
| Flag | SIGMA | F1804 |
| Myc | Cell Signaling Technologies | 2276S |
| V5 | Cell signaling Technilogies | 13202s |
| **Chemicals** | | |
| Dimethyl sulfoxide | SIGMA | D2650 |
| Actinomycin D | SIGMA | A1410-2MG |
| Camptothecin | SIGMA | C9911-100MG |
| Lipofectamin RNAimax | Invitrogen | 13778 |
| Lipofectamin 2000 | Invitrogen | 11668 |
| Protein G sepharose | GE Heathcare | GE17-0618-01 |
| Protein A sepharose | GE Heathcare | GE17-5280-01 |
| **Recombinant DNA** | | |
| pCMV Myc-TonEBP | This paper |  |
| pCMV Myc-Yc1 | This paper |  |
| pCMV Myc-Yc1 ΔRHD | This paper |  |
| pCMV FLAG-TonEBP | This paper |  |
| pCMV FLAG-Yc1 | This paper |  |
| pCMV FLAG-Yc1 (3M) | This paper |  |
| pCMV FLAG-Yc1 (5M) | This paper |  |
| pCMV GFP-Yc1 | This paper |  |
| pCMV GFP-Yc1 (3M) | This paper |  |
| pCMV GFP-Yc1 (5M) | This paper |  |
| pCMV GFP-Yc1 ΔRHD | This paper |  |
| pcDNA3.1 FLAG-METTL3 | This paper |  |
| pcDNA3.1 FLAG-METTL3 (-380) | This paper |  |
| pcDNA3.1 FLAG-METTL3 (-200) | This paper |  |
| pcDNA3.1 FLAG-METTL3  (381-580) | This paper |  |
| pCMV mcherry-METTL3 | This paper |  |
| ppyCAG V5-RNaseH1 | This paper |  |
| pCMV GFP-RNaseH1 | This paper |  |
| **Oligonucleotides** | | |
| Scramble siRNA | Invitrogen | N/A |
| TonEBP siRNA (Human) | Invitrogen | N/A |
| METTL3 siRNA (Human) | Invitrogen | N/A |
| **Experimental models: cell lines** | | |
| HEK293 | ATCC | CRL-1573 |
| U2OS | ATCC | HTB-96 |
| **Critical commercial assays** | | |
| Proximity ligase assay kit | SIGMA | DUO92101 |
| Click-IT EdU labeling kit | Invitrogen | C10634 |
| **Software and Algorithms** | | |
| Scaffold | Proteome software | http://www.proteomesoftware.com/products/scaffold/ |
| ImageJ | Processing scientific mutidimensional images | https://imagej.nih.gov/ij/ |
| Attovision | Imaging software | https://www.bioz.com/search/attovision%20software |

**Ⅲ. 2. Table S2. List of oligomers**

| **Name** | **Sequence** |
| --- | --- |
| COS_L | 5’-[Phos]-GGG CGG CGA CCT-3’ |
| COS_R | 5’-[Phos]-AGG TCG CCG CCC-3’ |
| Biotin_COS_L | 5’-[Phos]-GGG CGG CGA CCT-[Biotin]-3’ |
| Dig_COS_R | 5’-[Phos]-AGG TCG CCG CCC-[DIG]-3’ |
| Biotin_COS_R | 5’-[Phos]-AGG TCG CCG CCC-[Biotin]-3’ |
| Dig_COS_L | 5’-[Phos]-GGG CGG CGA CCT-[Dig]-3’ |
| R-loop oligo1* | 5’-[Cy3]-GCC AGG GAC GAG GTG AAC CTG CAG GTG GGC **GGC TAC TAC TTA GAT GTC ATC CGA GGC TTA T**TG GTA GAA TTC GGC AGC GTC ATG C GA CGG C-3’ |
| R-loop oligo2* | 5’-GCC GTC GCA TGA CGC TGC CGA ATT CTA CCA **CGC GAT TCA TAC CTG TCG TGC CAG CTG CTT T**GC CCA CCT GCA GGT TCA CCT CGT CCC TGG C-3’ |
| R-loop RNA | 5’-[Cy5]-GCA GCU GGC ACG ACA GGU AUG AAU C-3’ |
| D-loop DNA | 5’-GCA GCT GGC ACG ACA GGT ATG AAT C-3’ |
| Homoduplex | 5’-[Cy3]-GCC AGG GAC GAG GTG AAC CTG CAG GTG GGC AAA GCA GCT GGC ACG ACA GGT ATG AAT CGC GTG GTA GAA TTC GGC AGC GTC ATG CGA CGG C-3’ |
| Hybrid DNA | 5’-CCC ATA CCG TAT AAC CAT TTG GCT GTC CAA GCT CCG GGT-3’ |
| Hybrid RNA | 5’-[Cy5]-ACC CGG AGC UUG GAC AGC CAA AUG GUU AUA CGG UAU GGG-3’ |
| Fork1 | 5’-[Cy3]-GCC AGG GAC GAG GTG AAC CTG CAG GTG GGC GGC TAC TAC TTT TTT TTT TTT TTT TTT TTT TTT TTT TTT TTT TTT TTT T-3’ |
| Fork2 | 5’-TTT TTT TTT TTT TTT TTT TTT TTT TTT TTT TTT TTT TTT AGT AGT AGC CGC CCA CCT GCA GGT TCA CCT CGT CCC TGG C-3’ |
| Lambda_R-loop | 5’-[Phos]-TGC ATG CGG CCG CTC TTC CCA TGG TGC GAT TTT GTG GTT CCC ATA CCG TAT AAC CAT TTG GCT GTC CAA GCT CCG GGT GTT TGT TTT CCG CTC TTC CCA TGG TGC GAT CGC TCT TCG-3’ |
| Lambda_R-loop RNA | 5’-[Cy5]-ACC CGG AGC UUG GAC AGC CAA AUG GUU AUA CGG UAU GGG- 3’ |
| Lambda R-loop2 | 5’-[Phos]-TCG ACC CAG GGA CGA GGT GAA CCT GCA GGT GGG CGG CTA CTA CTT AGA TGT CAT CCG AGG CTT ATT GGT AGA ATT CGG CAG CGT CAT GCG ACG GG-3’ |
| Lambda R-loop2_comp | 5’-[Phos]-TCG ACC CGT CGC ATG ACG CTG CCG AAT TCT ACC ACG CGA TTC ATA CCT GTC GTG CCA GCT GCT TTG CCC ACC TGC AGG TTC ACC TCG TCC CTG GG-3’ |

* Bold represents bubble for R-loop.
